# Multi-omics analysis reveals glutathione metabolism-related immune suppression and constructs a prognostic model in lung adenocarcinoma

**DOI:** 10.3389/fimmu.2025.1608407

**Published:** 2025-07-02

**Authors:** Yuxiang Chi, Guoyuan Ma, Qiang Liu, Yunzhi Xiang, Defeng Liu, Jiajun Du

**Affiliations:** ^1^ Institute of Oncology, Shandong Provincial Hospital, Shandong University, Jinan, Shandong, China; ^2^ Cheeloo College of Medicine, Shandong University, Jinan, Shandong, China; ^3^ Department of Thoracic Surgery, Shandong Provincial Hospital Affiliated to Shandong First Medical University, Jinan, Shandong, China

**Keywords:** multi-omics, single-cell sequencing, glutathione metabolism, immunotherapy, prognostic model

## Abstract

**Background:**

Metabolic reprogramming within the tumor microenvironment plays a pivotal role in tumor progression and therapeutic responses. Nevertheless, the relationship between aberrant glutathione (GSH) metabolism and the immune microenvironment in lung adenocarcinoma, as well as its clinical implications, remains unclear.

**Methods:**

We leveraged genome-wide association study (GWAS) data and applied genetic causal analysis to evaluate the causal relationships among plasma 5-oxoproline levels, lung adenocarcinoma (LUAD) risk, and 731 immune phenotypes. We incorporated single-cell RNA sequencing data from LUAD to compare transcription factor activity, cell communication networks, and CD8^+^ T cell subset distributions across distinct GSH metabolic groups, followed by pseudotime analysis. Whole-transcriptome data from the TCGA database were analyzed for functional enrichment, immune infiltration, and immune functionality. Prognostic genes were identified using WGCNA and LASSO-Cox regression, and the expression was validated via qRT-PCR. Thereafter, immunotherapeutic efficacy and drug sensitivity were predicted using the TIDE platform and the oncoPredict package. A prognostic model was constructed to forecast patient survival, which was further validated in two independent GEO datasets.

**Results:**

Genetic causal analysis indicated a positive correlation between plasma 5-oxoproline levels and LUAD risk. ScRNA-seq analysis revealed an increased proportion of exhausted CD8^+^ T cells in the high GSH metabolic group, accompanied by altered transcription factor activity and distinct cell communication patterns. Furthermore, whole-transcriptome data analysis demonstrated that patients with a high metabolic phenotype exhibited significantly diminished immune functionality and overall immune infiltration. Using WGCNA and LASSO-Cox regression, we ultimately identified three key genes (LCAL1, RHOV, and MARCHF4) and generated a gene risk score. This score effectively predicts both immunotherapy response and drug sensitivity. qRT-PCR confirmed the upregulation of MARCHF4 in LUAD cells. In addition, stratification by gene risk scores revealed significant differences in immune cell infiltration, immunotherapeutic response, and drug sensitivity. The nomogram model demonstrated strong predictive accuracy in both the TCGA cohort and two independent GEO validation datasets.

**Conclusions:**

GSH metabolic reprogramming may suppress antitumor immunity by modulating transcription factor activity, remodeling cell communication networks, and regulating CD8+ T cells. The prognostic risk model developed herein effectively predicts immunotherapeutic response, drug sensitivity, and overall survival in patients with LUAD.

## 1 Introduction

Among lung cancers, lung adenocarcinoma (LUAD) represents the predominant histological subtype, with an exceedingly complex pathogenesis that encompasses genetic, environmental, and lifestyle factors (1, 2). The development and progression of tumors depend not only on intrinsic genetic mutations and molecular abnormalities but also on the surrounding tumor microenvironment (TME) (3). Tumor cells frequently employ metabolic reprogramming to adapt to the evolving conditions of the TME, thereby facilitating their survival (3, 4).

Within the intricate process of tumor metabolic reprogramming, the regulation of redox balance in tumor cells is critically important (5). Glutathione (GSH), the primary intracellular antioxidant, is indispensable for scavenging reactive oxygen species (ROS) and maintaining redox homeostasis (6). Studies have shown that its levels are markedly elevated in various human tumors, including those in the head and neck, breast, ovary, colorectum, and lung, compared with normal tissues (7–9). Elevated GSH levels enable tumor cells to neutralize harmful peroxides and sustain redox balance, thereby creating a microenvironment that favors rapid cellular proliferation (7, 10). Consequently, the reprogramming of the GSH metabolic pathway is essential for tumor survival and progression.

The metabolism of GSH is primarily dependent on the classical γ-glutamyl cycle (10, 11). When glutathione synthesis is impaired or depleted, the intermediate 5-oxoproline can accumulate abnormally (10, 12, 13). Clinical reports have documented that impaired glutathione metabolism leads to the accumulation of 5-oxoproline, which in turn precipitates metabolic acidosis in patients (12, 14). Therefore, the concentration of 5-oxoproline can, to some extent, reflect the activity and integrity of the glutathione cycle. Based on this evidence, analyzing the metabolite 5-oxoproline, which is an indicator of GSH levels, may elucidate its relationship with tumorigenesis and progression.

An increasing number of studies indicate that tumor metabolic reprogramming significantly impacts antitumor immune responses (4, 15–17). Within the tumor microenvironment, intense competition for nutrients, continuous accumulation of metabolic waste, and complex alterations in metabolic signals collectively affect immune cell infiltration, survival, and function (16–18). In particular, T lymphocytes, which are key effector cells in antitumor immunity, rely heavily on metabolic pathways to support their proliferation, differentiation, and cytotoxic activity (19, 20). Accordingly, a detailed analysis of the interplay between aberrant glutathione metabolism and immune suppression in tumors is of considerable theoretical and practical significance for refining current therapeutic strategies.

Given the close association between glutathione metabolism, the tumor microenvironment, and immune function, this form of metabolic reprogramming may substantially influence treatment responses in lung adenocarcinoma. In the field of immunotherapy, although immune checkpoint inhibitors such as PD-1/PD-L1 blockers have demonstrated a degree of efficacy, only approximately 20–30% of patients exhibit a favorable response (21). This variability may be attributable to differences in the tumor metabolic microenvironment, with glutathione metabolism emerging as a key factor (22, 23). Moreover, glutathione levels may affect tumor cell sensitivity to chemotherapy and targeted therapies (24). For example, elevated GSH can attenuate the cytotoxic effects of chemotherapeutic agents, such as platinum-based and anthracycline drugs, by neutralizing excess reactive oxygen species generated during treatment, thereby promoting drug resistance (24). Additionally, glutathione participates in drug metabolism and efflux, forming conjugates that facilitate drug excretion and lower intracellular drug concentrations (24). Hence, analyzing molecular features associated with glutathione metabolism holds promise for developing more precise predictive tools for drug sensitivity.

Although the TNM staging system is widely used for risk stratification in lung adenocarcinoma, patients within the same stage can experience disparate clinical outcomes, highlighting the critical influence of tumor-specific biological characteristics on prognosis. As an integral aspect of tumor metabolic reprogramming, glutathione metabolism and the expression of its related genes may serve as an indicator of a tumor’s invasive and progressive potential (7, 24). Recent studies have demonstrated that the expression of various redox-related genes is closely associated with patient survival (25). By integrating molecular features related to glutathione metabolism, it is possible to develop prognostic models that not only enhance the accuracy of survival predictions but also offer a more comprehensive molecular basis for evaluating immunotherapy responses and drug sensitivity in lung adenocarcinoma, thereby facilitating personalized treatment decisions.

Against this backdrop, this investigation represents the first comprehensive integration of multi-omics data, combining genetic causal inference, single-cell RNA sequencing, and global transcriptome analysis to explore the relationship between glutathione metabolic dysregulation and tumor immunosuppression in LUAD. Furthermore, the study develops a prognostic risk model based on identified glutathione metabolism-related differential genes to evaluate its potential applications in predicting immunotherapy efficacy, determining drug sensitivity, and stratifying prognostic risk. This approach provides novel theoretical foundations and clinical decision-making tools for precision treatment of lung adenocarcinoma patients.

## 2 Materials and methods

### 2.1 Genome-wide association study data acquisition

We obtained the GWAS dataset for 5-oxoproline levels (ebi-a-GCST90200280) from the GWAS Catalog, originating from the Canadian Longitudinal Study on Aging (CLSA) (26, 27). We acquired comprehensive GWAS data for 731 immune phenotypes from the largest study to date, which involved 3,757 Italian Sardinian residents (accession numbers ebi-a-GCST90001391 to ebi-aGCST90002121) (28). We obtained data for lung adenocarcinoma from FinnGen R11 (29). On June 24, 2024, the FinnGen project released the 11th version (R11), including genotype and health data for 453,733 participants, covering 21,311,942 variant sites and 2,447 disease endpoints (phenotypes). Compared to previous versions, R11 offers increased sample size, more phenotypes, and broader genetic variant coverage. The populations used for the GWAS data analysis were non−overlapping.

### 2.2 Instrumental variables selection

We employed a multi-step filtering approach to identify valid instrumental variables for genetic causal analysis. First, Single Nucleotide Polymorphisms (SNPs) showing significant associations were identified using a P-value threshold of less than 5 × 10^-6^. Second, to ensure independence among genetic variants, we conducted linkage disequilibrium (LD) clumping using PLINK with a 10,000 kb window and an r² threshold of 0.001, referencing the European population from the 1000 Genomes Project. Finally, to mitigate bias from weak instruments, we calculated the F-statistic for each remaining SNP and retained only those with values greater than 10.

### 2.3 Genetic instrument-based mediation analysis

We conducted genetic causal analyses using the TwoSampleMR package. We applied five genetic causal inference methods: inverse variance weighted (IVW), MR-Egger, weighted median, weighted mode, and simple mode. Among these, IVW was the primary method (30, 31).

We performed mediation analysis using genetic instruments to assess whether immune cells mediate the effect of 5-oxoproline on lung adenocarcinoma (32, 33). First, we estimated the total effect of 5-oxoproline on lung adenocarcinoma (Beta 0) using conventional genetic instrumental variable analysis. Subsequently, the total effect was decomposed into a direct effect (Beta 3) and an indirect effect mediated by immune cells. The indirect effect was calculated as the product of the effect of 5-oxoproline on immune cells (Beta 1) and the effect of immune cells on lung adenocarcinoma (Beta 2). To assess the significance of the mediation, we calculated the ratio of the indirect effect to the total effect, known as the mediation proportion.

### 2.4 Sensitivity analysis

Sensitivity analyses included tests for heterogeneity, pleiotropy, leave-one-out analysis, and MR-PRESSO. The consistency of the genetic instrument effects was assessed using the IVW method and MR-Egger regression. A p-value < 0.05 in the heterogeneity test indicates significant heterogeneity in the analysis. The MR-Egger intercept test was used to detect pleiotropy and evaluate the robustness of the results. A p-value < 0.05 indicates pleiotropy. The leave-one-out method was employed to identify outliers by sequentially removing each SNP and assessing the stability of the results. MR-PRESSO offers a global test to evaluate the significance of pleiotropy.

### 2.5 Single-cell data source and preprocessing

We integrated two independent scRNA-seq datasets from LUAD patient samples (GSE131907 and PRJNA632939) using the Seurat package (34–36). Initial quality control was performed using the following criteria: cells with gene counts between 300 and 5,000; unique molecular identifiers (UMIs) between 500 and 40,000; mitochondrial gene proportion below 8%; and hemoglobin gene proportion below 3%. Genes expressed in fewer than three cells were excluded. After quality control, each cell’s total UMI count was normalized to 10,000 and then log-transformed. The top 2,000 highly variable genes were identified using a variance-stabilizing transformation.

To minimize technical variation and batch effects between datasets, we applied an integrated strategy. We first normalized the data and performed principal component analysis (PCA). We then conducted batch correction using the Harmony algorithm, with dataset origin as the batch variable. Ultimately, we combined data from two datasets, encompassing 41 lung tissue samples from 25 patients, resulting in a single-cell cohort of 150,063 cells for further analysis.

### 2.6 Cell-clustering and annotation

LUAD samples were normalized to identify the top 2,000 highly variable genes. Subsequently, the data underwent scaling and initial dimensionality reduction. A neighborhood graph was then constructed using the first six principal components, and cells were clustered at a resolution of 0.5. We manually annotated each cluster based on known cell marker gene expression in combination with the SingleR algorithm (37). To improve the accuracy of cell type annotation in single-cell RNA sequencing data, we employed the DeepSeek large−model Application Programming Interface (API) as an ancillary tool. We also generated a DotPlot of marker genes to display characteristic gene expression across cell subpopulations, aiding in further validation of annotation accuracy.

### 2.7 Single-cell sequencing data glutathione metabolism grouping

We downloaded the Kyoto Encyclopedia of Genes and Genomes(KEGG) glutathione metabolism GMT file from Molecular Signatures Database (MSigDB) (https://www.gsea-msigdb.org/gsea/msigdb/) and loaded the relevant genes using the read.gmt function. We used the AddModuleScore function in the Seurat package to calculate a module score for each cell. We applied the extracted gene set as a feature list for scoring, generating a metabolic module score for each cell that reflects the overall activity of the glutathione metabolism pathway. We then compared glutathione metabolism scores across different sample types, such as normal tissues and LUAD samples. We grouped tumor samples into high GSH metabolism group and low GSH metabolism group based on the median glutathione metabolism module score.

### 2.8 Transcription factor activity prediction

To predict transcription factor activity in LUAD cells, we utilized the human transcription factor regulatory network from the DoRothEA database, selecting transcription factors and target genes with confidence levels A, B, and C (38). We inferred transcription factor activity using the Virtual Inference of Protein-activity by Enriched Regulon analysis (VIPER) algorithm in the decoupleR package. After integrating inference results with cell population data, we calculated the mean activity and standard deviation of each transcription factor across different cell types. We identified the 20 transcription factors with the greatest activity changes between high and low GSH metabolism groups, illustrating their expression patterns across cell populations. Furthermore, we highlighted immune-related transcription factors (e.g., BATF, BCL6, EOMES) to reveal their potential roles in immune regulation and T cell function.

### 2.9 Cell communication

We conducted cell communication analysis on cells with high GSH metabolism group and low GSH metabolism group in LUAD using the CellChat package (39, 40). We estimated communication probabilities for ligand–receptor pairs using a truncated mean and retained only those interactions detected in at least ten cells to ensure robust results. Thereafter, we aggregated and categorized these interactions to build intercellular communication networks, comparing network strength and pathway activities between the two metabolic groups to identify pathways with significantly altered activity. We calculated network centrality metrics for each CellChat object to identify senders and receivers within the communication network, highlighting changes in communication roles across different metabolic groups.

### 2.10 T cell-clustering and annotation

To further analyze CD8+ T cell subsets in lung adenocarcinoma samples, we first extracted the T cell subset from the manually annotated Seurat object, isolating T cells with CD8A expression greater than 50% as CD8+ T cells. We preprocessed the CD8^+^ T cell subset by normalizing the data, identifying highly variable genes, and correcting for batch effects. We then performed principal component analysis and, guided by the elbow plot, selected the first six components for subsequent neighbor‐graph construction and clustering. Finally, we clustered cells at a resolution of 0.5. Based on prior research and expression patterns of cell marker genes, we manually annotated each cluster as effector CD8+ T cells, memory CD8+ T cells, naive CD8+ T cells, and exhausted CD8+ T cells (41, 42). Furthermore, we generated a DotPlot of marker genes to display characteristic gene expression across CD8+ T cell subsets, serving to further validate the accuracy of the annotations.

### 2.11 Trajectory analysis of single cells

To investigate the developmental trajectory of CD8+ T cells, we performed pseudotime analysis using the Monocle2 package (43). To avoid batch effects, we extracted and analyzed the CD8+ T cell subset from “GSE131907.” To assess the functional state of T cells, we calculated the Naïve Score based on the expression of a characteristic gene set including SELL, LEF1, and CCR7. We conducted dimensionality reduction using the DDRTree algorithm with a maximum component number set to 2. To establish the starting point of the trajectory, we designated cells with the highest initial Naïve Score as the root state, based on which we ordered cells to calculate pseudotime, reconstructing the developmental trajectory of CD8+ T cells. Subsequently, we calculated the proportions of each CD8+ T cell subset and compared their glutathione metabolism scores.

### 2.12 Bulk RNA-seq data source

We retrieved lung adenocarcinoma transcriptome profiles and associated clinical data from The Cancer Genome Atlas (TCGA) database. Additionally, two independent lung adenocarcinoma transcriptome datasets (GSE31210 and GSE13213) were obtained from the Gene Expression Omnibus (GEO) database for validation purposes (44, 45). Gene annotation was performed using the biomaRt package in R, during which unannotated genes were filtered out. Raw count data were normalized using the edgeR package. To ensure data quality, samples lacking survival information, those with survival times shorter than one month, and those with missing age, pathological stage, or expression data were excluded. Ultimately, the TCGA lung adenocarcinoma cohort (n = 497) served as the training set for data analysis and model construction, while the GEO datasets GSE31210 (n = 226) and GSE13213 (n = 117) were used as independent validation sets to evaluate external predictive performance.

### 2.13 Bulk RNA transcriptomic glutathione metabolism grouping

In this study, a glutathione metabolism gene set, which was previously downloaded from the MSigDB database, was employed together with the GSVA package (46). The single-sample gene set enrichment analysis (ssGSEA) method was used to compute a glutathione metabolism score for each sample. Based on these scores, lung adenocarcinoma samples from the TCGA dataset were divided into high GSH metabolism group and low GSH metabolism group using the median as the threshold. To further validate the relationship between metabolic profiles and transcript copy numbers, we performed a comprehensive correlation analysis between GSH-related genes and the calculated GSH score. Based on TCGA-LUAD data and the GSH score computed by ssGSEA, we extracted the REACTOME_GLUTATHIONE_SYNTHESIS_AND_RECYCLING gene set. For each gene, we conducted a Pearson correlation test between its expression level and the GSH score, applying the Benjamini–Hochberg method to adjust for multiple testing.

To investigate the differences in central carbon metabolic flux between high- and low-GSH metabolism groups, we analyzed four key metabolic pathways obtained from the MSigDB using KEGG gene sets. The selected pathways included (1): Glycolysis/Gluconeogenesis (KEGG_GLYCOLYSIS_GLUCONEOGENESIS), (2) Citrate cycle/TCA cycle (KEGG_CITRATE_CYCLE_TCA_CYCLE), (3) Pentose phosphate pathway (KEGG_PENTOSE_PHOSPHATE_PATHWAY), and (4) Glycine, serine, and threonine metabolism (KEGG_GLYCINE_SERINE_AND_THREONINE_METABOLISM). We employed the GSVA package to perform single-sample gene set enrichment analysis and calculate a pathway activity score for each sample. We then used the Wilcoxon rank-sum test to compare these pathway activity scores between the high GSH and low GSH metabolism groups.

### 2.14 Differential expression analysis

For the differential expression analysis, the edgeR package was primarily employed. We identified differentially expressed genes (DEGs) by applying a false discovery rate (FDR) cutoff of <0.05 and an absolute log_2_ fold change threshold of >1.

### 2.15 Functional enrichment analysis

We performed Gene Ontology (GO) enrichment analysis and gene set enrichment analysis (GSEA) of differentially expressed genes using the clusterProfiler package. GO enrichment was conducted separately for upregulated and downregulated genes, encompassing the three categories of biological process (BP), cellular component (CC), and molecular function (MF). Only GO terms with an adjusted p-value below 0.05 were considered, and the top five most significant entries from each category were presented. Furthermore, a Hallmark gene set, which comprises 50 gene sets related to key tumor biological features, was downloaded from the MSigDB database. GSEA was performed on the DEGs, with a false discovery rate (FDR) < 0.05 set as the threshold for determining pathway significance.

### 2.16 Analysis of immune infiltration, immune function and mutation in metabolic subgroups

The xCell package was utilized to assess immune infiltration in the TCGA lung adenocarcinoma cohort across different glutathione metabolism groups (47). This evaluation focused on 20 primary immune cell types, including B cells, CD4^+^ T cells and their subsets, CD8^+^ T cells and their subsets, Th1/Th2 cells, regulatory T cells, macrophages, mast cells, monocytes, and natural killer (NK) cells, along with an overall immune score.

We retrieved 29 immune-related gene sets from the MSigDB database to assess the influence of glutathione metabolism on immune function in lung adenocarcinoma. The ssGSEA algorithm within the GSVA package was then applied to assess the immune activity of each sample. Evaluated immune functions included co-stimulatory and co-inhibitory signals of antigen-presenting cells and T cells, checkpoint molecule expression, cytotoxic activity, inflammatory response, and various cytokine signaling pathways.

To investigate the mutation landscape associated with GSH metabolism, we downloaded somatic mutation data for TCGA-LUAD samples. The mutation data were processed using the maftools package in R. The oncoplot function was used to generate waterfall plots visualizing the top 20 most frequently mutated genes in each metabolic group. Fisher’s exact test was employed to compare statistical differences in mutation frequencies between the groups.

### 2.17 Weighted gene co-expression network analysis

WGCNA, through its weighted network calculations, systematically identified co-expression modules significantly associated with glutathione metabolism scores, thereby reducing bias inherent in single differential analysis approaches. Based on the TCGA lung adenocarcinoma training dataset, we constructed a WGCNA to identify gene modules associated with GSH metabolism (48). Standardized expression data were used to build an expression matrix, and sample clustering was performed to remove outliers. Using the R package WGCNA, various soft-thresholding powers (ranging from 1 to 20) were evaluated, and a power of 6 was selected based on the scale-free topology fit index for network construction. Hierarchical clustering and dynamic tree cutting (with a minimum module size of 80) were applied to identify gene co-expression modules, and modules with a similarity threshold of 0.25 were merged. Subsequently, module eigengenes were correlated with the GSH metabolic status, and modules with an absolute correlation coefficient (|r|) > 0.25 and a p-value < 0.05 were selected. The candidate core gene set was then obtained by intersecting the genes in these modules with the differentially expressed genes.

### 2.18 LASSO-Cox regression for prognostic gene selection

LASSO-Cox regression introduces L1 regularization to high-dimensional transcriptomic data, simultaneously addressing multicollinearity while preventing overfitting, thus enhancing model robustness. Its coefficient shrinkage mechanism automatically selects independent prognostic markers from numerous candidate genes, simplifying model structure and improving clinical interpretability. We used the survival package to conduct univariate Cox proportional hazards regression on core WGCNA module genes and assess their association with overall survival in lung adenocarcinoma patients. To address multiple testing, p-values were adjusted using the FDR correction, and genes with an adjusted FDR < 0.05 were selected as candidates for further analysis.

We used the glmnet package to perform least absolute shrinkage and selection operator (LASSO) regression, refining prognostic gene selection and reducing overfitting risk. We determined the optimal penalty parameter λ by ten-fold cross-validation and selected genes with non-zero coefficients at the λ value that minimized cross-validation error as final prognostic marker candidates. LASSO coefficient paths and cross-validation curves were generated to visually illustrate the feature selection process.

We integrated the LASSO-selected genes into a multivariable Cox regression model to evaluate their independent impact on survival in lung adenocarcinoma patients. Genes with p < 0.05 were deemed independent prognostic markers.

### 2.19 Quantitative real-time PCR experiments

Normal lung epithelial cell line BEAS-2B and lung adenocarcinoma cell line H2030 were obtained from Shanghai Fuheng Biological Company. Both cell types were cultured in RPMI 1640 medium supplemented with 10% fetal bovine serum and 1% penicillin-streptomycin under standard conditions (37°C, 5% CO_2_). Total RNA was extracted using Nucleozol according to the manufacturer’s instructions, and reverse transcription was performed with Prime Script RTase (Novozan) as recommended. mRNA expression levels were subsequently measured by qRT-PCR using SYBR Green (AG) following the manufacturer’s protocol. The experiment was independently repeated three times.

The primer sequences for the prognostic model gene MARCHF4 were as follows:

Forward primer: GCTACGGGATGTATGGCTTCA.
Reverse primer: TCCTCCAGGTCTTTTGTCTTGTC.

### 2.20 Construction of metabolic risk score and grouping

We developed a prognostic risk score model for lung adenocarcinoma based on three genes identified during preliminary screening. We calculated each patient’s risk score as a weighted sum of gene expression values using coefficients from the Cox proportional hazards regression model:


Gene risk score = ∑(Expi*Coefi).

We used the survivalROC package to generate ROC curves for predicting 36-month survival and selected the optimal cutoff by maximizing the Youden index. We then stratified TCGA lung adenocarcinoma patients into high and low gene score groups and compared their survival using Kaplan–Meier analysis.

### 2.21 Analysis of immune infiltration and immune function in metabolic risk groups

Using the xCell package, immune infiltration was assessed across different gene score groups within the TCGA lung adenocarcinoma cohort. To examine differences in immune function between these groups, 29 immune function gene sets were downloaded from the MSigDB database and the ssGSEA algorithm from the GSVA package was employed to calculate an immune function score for each sample.

### 2.22 Immunotherapy and drug sensitivity prediction

Immune checkpoint genes were downloaded from the ImmPort database to compare differences in immune checkpoint gene expression among the different gene score groups. The Tumor Immune Dysfunction and Exclusion (TIDE) web tool was used to evaluate differences in immunotherapy response between the groups (49). TIDE is a comprehensive scoring system that estimates tumor immune escape mechanisms and predicts patient response to immune checkpoint inhibitors. By uploading the expression data from the TCGA lung adenocarcinoma dataset to the TIDE website, each sample’s overall TIDE score, immune exclusion score, and T cell dysfunction score as well as predicted immunotherapy response were obtained. Furthermore, the oncoPredict package was utilized to predict the sensitivity of patients in different gene score groups to chemotherapeutic and targeted agents (50). The drug sensitivity predictions were based on a training model derived from the Cancer Drug Sensitivity Genomics 2 (GDSC2) database, which includes cell line expression profiles and corresponding IC50 values (the concentration of drug required to inhibit 50% of cell growth). Based on the results, drugs were categorized into three groups: those to which the low gene score group was more sensitive (green), those to which the high gene score group was more sensitive (red), and those with no statistically significant difference (blue).

### 2.23 Construction of a prognostic nomogram

Using the TCGA lung adenocarcinoma dataset, a prognostic nomogram was developed incorporating age, tumor stage, and gene score group. Patients were stratified into age groups (<50, 50–60, 60–70, and >70 years) based on clinical relevance, and tumor stage was simplified into I, II, III, and IV according to the AJCC staging system. We used the survival package to conduct univariate and multivariate Cox proportional hazards regression analyses. Univariate Cox analysis evaluated the association between each independent variable and patient survival outcomes, calculating hazard ratios (HRs) and 95% confidence intervals (95% CI). Variables significant in the univariate analysis were then included in the multivariate model to assess their independent impact on survival after adjusting for other covariates. To construct a user-friendly prognostic tool, the rms package was employed to create a nomogram based on the multivariate Cox regression model, visually representing the contribution of each independent prognostic factor to the prediction of 1-, 3-, and 5-year overall survival.

### 2.24 Validation of the prognostic model

The timeROC package was used to generate ROC curves for 1-, 3-, and 5-year survival in both the TCGA training set and the GEO validation set. The area under the ROC curve (AUC) was used as an indicator of model performance, with higher AUC values indicating better predictive accuracy. Kaplan–Meier survival analysis was performed to evaluate overall survival differences between high-risk and low-risk patient groups. Both the TCGA training set and the GEO validation set were stratified into high- and low-risk groups based on the median value of the risk score model, and survival curves were generated using the survival and survminer packages in R. Calibration curves for 1-, 3-, and 5-year survival were produced using the rms package. Model calibration was internally validated via bootstrap resampling (1,000 iterations) to assess the stability of the model. Additionally, decision curve analysis (DCA) was conducted using the ggDCA package to evaluate the clinical utility of the model at different risk thresholds. In the DCA plot, the x-axis represents the risk threshold and the y-axis represents the net benefit, with higher curves indicating greater clinical decision-making value at the corresponding risk thresholds.

### 2.25 Statistical analysis

The statistical analysis of this study was performed using R4.4.1 software. All quantitative data were first assessed for normality using the Shapiro-Wilk test. We compared two groups of normally distributed data using Student’s t-test. We applied the Mann–Whitney U test to data that did not follow a normal distribution. When data met normality and homogeneity of variance, we performed one-way analysis of variance for comparisons across more than two groups. When these assumptions were violated, we used the Kruskal–Wallis H test. All tests were conducted as two-tailed analyses, and P < 0.05 was considered as the significance threshold. *P < 0.05, **P < 0.01, and ***P < 0.001.

## 3 Results

### 3.1 Genetic causal analysis

**Figure 1:**
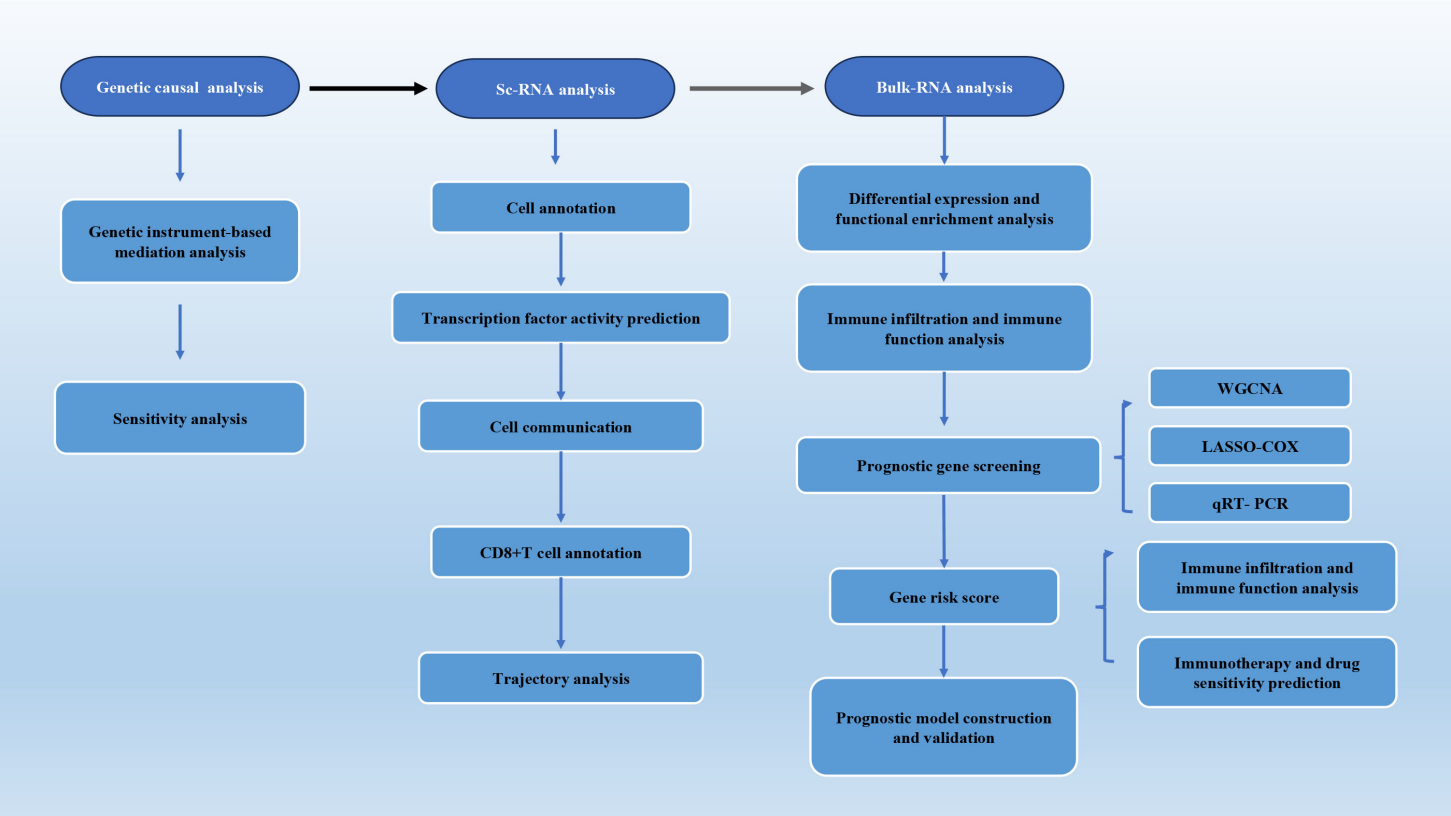
The overall design of our study.

**Figure 2:**
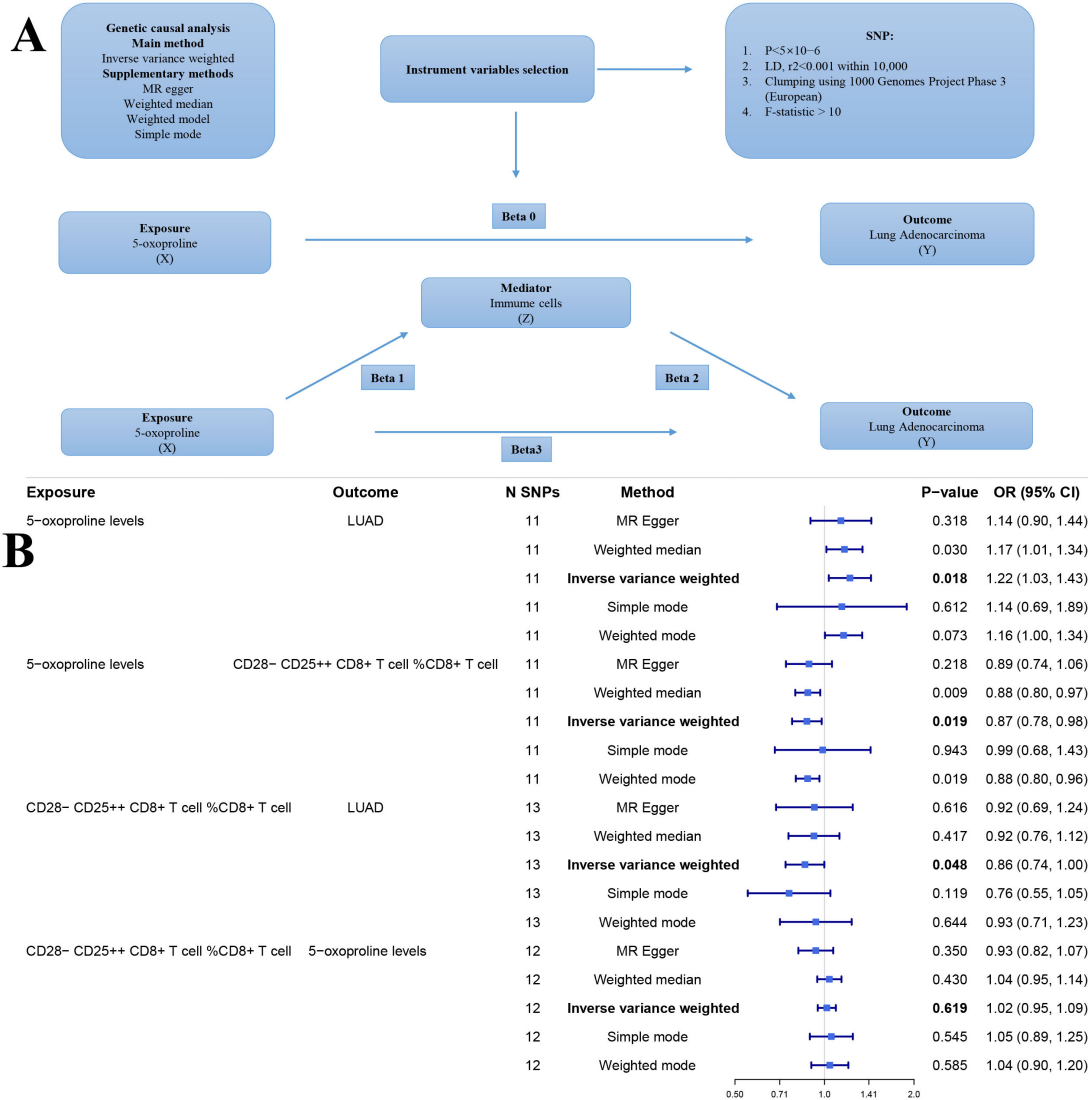
**(A)** Workflow of the genetic causal analysis. X (Exposure): 5-oxoproline; Y (Outcome): lung adenocarcinoma (LUAD); Z (Mediator): immune cell. **(B)** Forest plot of genetic instrument-based mediation analysis results. nsnp, number of single nucleotide polymorphisms; pval, p-value; OR, odds ratio.

The overall design of our study is depicted in the flowchart (**Figure 1**). **Figure 2A** illustrates the workflow for the genetic instrument-based mediation analysis. Initially, we performed a genetic causal analysis to assess the causal influence of 5−oxoproline on LUAD risk. The IVW estimate revealed a positive association between genetically predicted 5−oxoproline levels and LUAD susceptibility (IVW: OR = 1.22, 95% CI: 1.03–1.43, P < 0.05) (**Figure 2B**). Thereafter, to comprehensively explore the contribution of immune cell subsets to LUAD pathogenesis, we undertook separate genetic causal analyses for 731 distinct immune phenotypes. Employing the same IVW approach, 37 immune traits were found to be significantly associated with LUAD risk (P < 0.05) (**Supplementary Table 1**). Notably, CD28^-^CD25^++^ CD8^+^ T cells exhibited a protective effect against LUAD (IVW: OR = 0.86, 95% CI: 0.74–1, P < 0.05) (**Figure 2B**). Based on this result, we then investigated whether 5−oxoproline levels causally influence the abundance of CD28^-^CD25^++^ CD8^+^ T cells. The IVW analysis demonstrated a significant inverse relationship (IVW: OR = 0.87, 95% CI: 0.78–0.98, P < 0.05) (**Figure 2B**). To validate and further characterize this bidirectional link, we conducted a reverse genetic causal analysis to determine if CD28^-^CD25^++^ CD8^+^ T cells might, in turn, modulate 5−oxoproline levels. No significant effect was detected in the reverse direction (P= 0.619) (**Figure 2B**). Collectively, these findings suggest that elevated 5−oxoproline may promote LUAD development by reducing the proportion of CD28^-^CD25^++^ CD8^+^ T cells. Mediation analysis estimated a direct effect of 0.176 and an indirect effect of 0.021, with the mediator accounting for 11.9% of the total effect.

**Figure 3:**
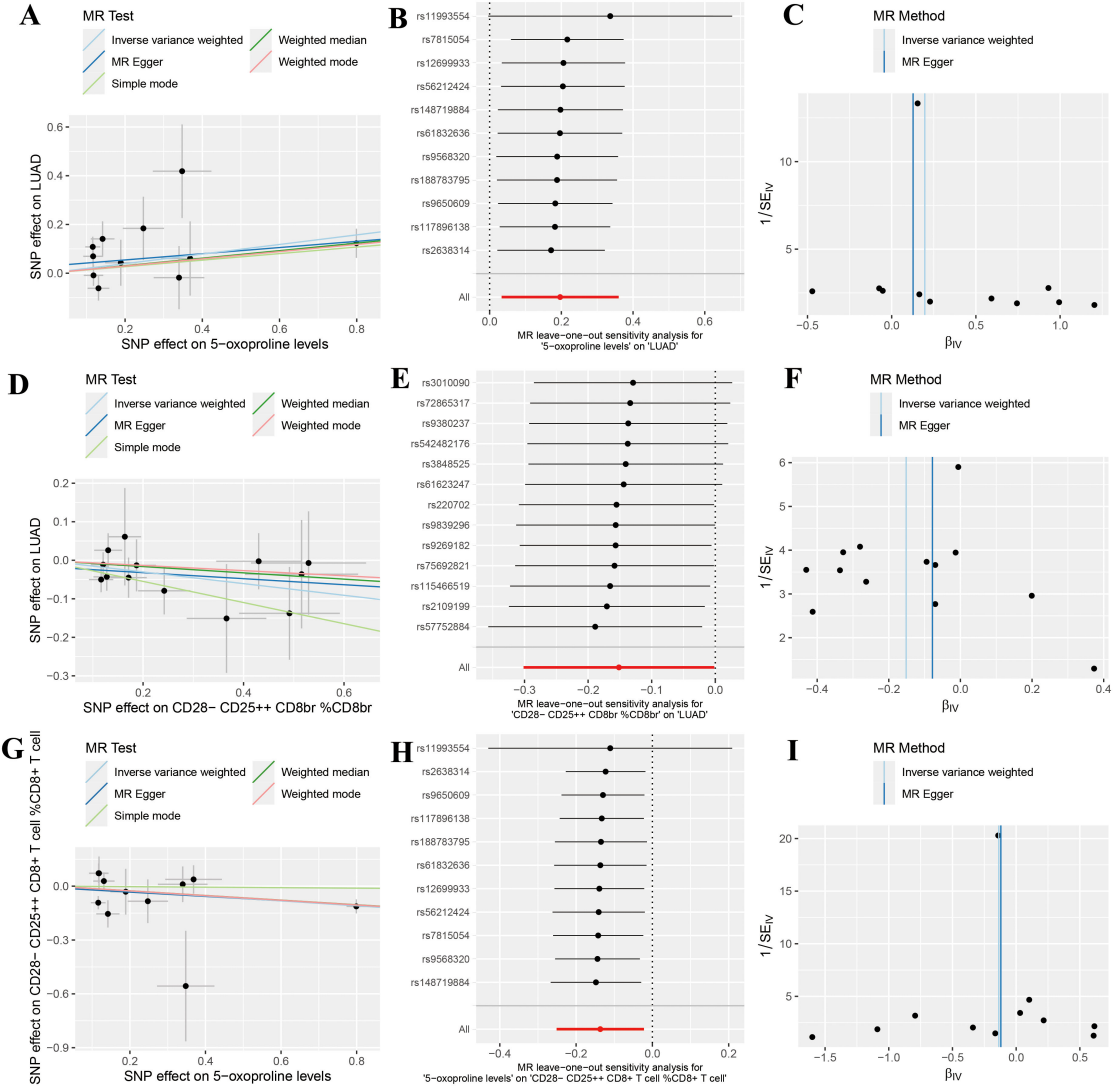
Sensitivity analysis results indicating no significant heterogeneity or pleiotropy in positive findings. **(A)** Scatter plot analyzing the relationship between SNP effects and the association of 5-oxoproline levels with lung adenocarcinoma (LUAD) risk using five genetic instrumental variable analysis methods (Inverse Variance Weighted, MR Egger, Simple Mode, Weighted Median, and Weighted Mode); **(B)** Forest plot of the leave-one-out sensitivity analysis for the association between 5-oxoproline levels and LUAD risk; **(C)** Funnel plot of the genetic instrumental variable analysis for the association between 5-oxoproline levels and LUAD risk; **(D)** Scatter plot analyzing the relationship between SNP effects and the association of CD28-CD25^++^CD8br %CD8br with LUAD risk using five genetic instrumental variable analysis methods; **(E)** Forest plot of the leave-one-out sensitivity analysis for the association between CD28-CD25^++^CD8br %CD8br and LUAD risk; **(F)** Funnel plot of the genetic instrumental variable analysis for the association between CD28-CD25^++^CD8br %CD8br and LUAD risk; **(G)** Scatter plot analyzing the relationship between SNP effects and the association of CD28-CD25^+^CD8^+^T cells %CD8^+^T cells with 5-oxoproline levels using five genetic instrumental variable methods; **(H)** Forest plot of the leave-one-out sensitivity analysis for the association between 5-oxoproline levels and CD28-CD25^+^CD8^+^T cells %CD8^+^T cells; **(I)** Funnel plot of the genetic instrumental variable analysis for the association between 5-oxoproline levels and CD28-CD25^+^CD8^+^T cells %CD8^+^T cells.

Sensitivity analyses revealed no evidence of heterogeneity (P > 0.05) or pleiotropy (P > 0.05), further affirming the robustness and reliability of the causal inference (**Supplementary Tables 2**, **3**). Scatter plots demonstrated consistent trends across five genetic causal analysis methods (**Figures 3A, D, G**). Leave-one-out analyses confirmed that the exclusion of any single SNP did not significantly alter the overall effect estimates (**Figures 3B, E, H**). Moreover, funnel plots exhibited a relatively symmetrical distribution, indicating no marked horizontal pleiotropy (**Figures 3C, F, I**). Overall, these results support the validity and homogeneity of the genetic instrumental variable analysis findings. Additionally, MR-PRESSO analysis did not detect any outlier SNPs or significant pleiotropy.

### 3.2 Identification and annotation of lung adenocarcinoma cell subpopulations

**Figure 4:**
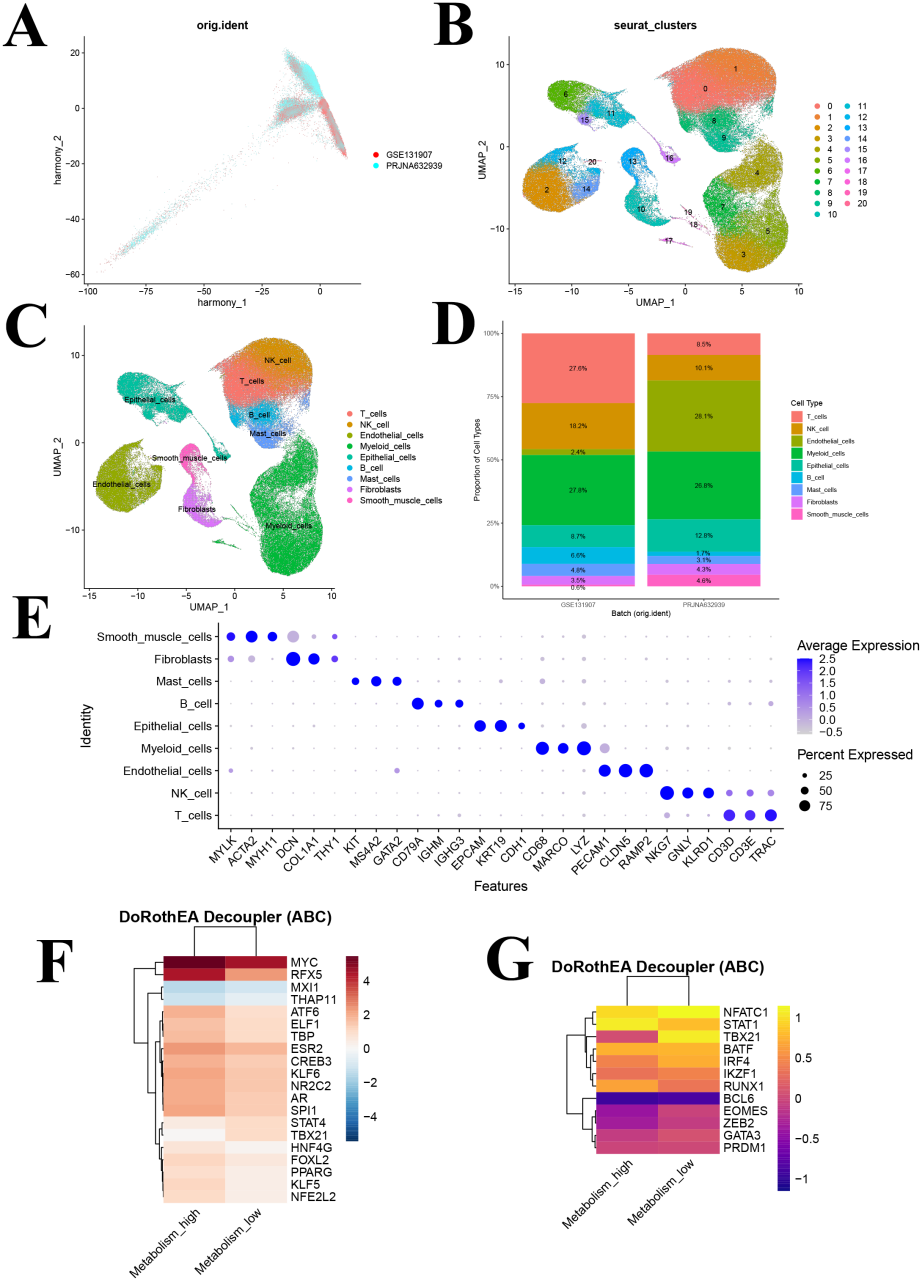
Identification of cell clusters based on scRNA-seq data from lung adenocarcinoma (LUAD) patients and transcription factors analysis. **(A)** PCA plot of single-cell RNA-seq data after Harmony batch correction; **(B)** UMAP of 21 cell clusters; **(C)** UMAP following cell annotation; **(D)** Cell type distribution across different batches; **(E)** Dot plot of marker genes for each cell type; **(F)** Heatmap showing the activity of the top 20 most variable transcription factors across metabolism groups. **(G)** Heatmap depicting activity patterns of 12 immune-related transcription factors across metabolism groups. UMAP, uniform manifold approximation and projection.

Following rigorous quality control, normalization, and batch effect correction, we performed principal component analysis, and the results showed that cells from the two sample sources were highly intermixed in the two-dimensional coordinate space, with no distinct clustering or separation observed (**Figure 4A**). An independent cell clustering analysis of lung adenocarcinoma samples identified 21 distinct cell clusters (**Figure 4B**). Based on the expression patterns of canonical marker genes, these clusters were annotated into nine major cell types: T cells, NK cells, endothelial cells, myeloid cells, epithelial cells, B cells, mast cells, fibroblasts, and smooth muscle cells (**Figure 4C**). Subsequently, we compared the composition of major cell types between the two sample sources (**Figure 4D**). Identification was supported by specific marker gene expression, including T cells (CD3D, CD3E, TRAC), NK cells (NKG7, GNLY, KLRD1), endothelial cells (PECAM1, CLDN5, RAMP2), myeloid cells (CD68, MARCO, LYZ), epithelial cells (EPCAM, KRT19, CDH1), B cells (CD79A, IGHM, IGHG3), mast cells (KIT, MSA42, GATA2), fibroblasts (DCN, COL1A1, THY1), and smooth muscle cells (MYLK, ACTA2, MYH11) (**Figure 4E**).

### 3.3 Transcription factor activity analysis reveals immune regulatory differences

Utilizing the DoRothEA database and the VIPER algorithm, we performed a transcription factor activity analysis. The 20 most significantly altered transcription factors were identified, with elevated activity observed for MYC, RFX5, ATF6, and NFE2L2 in the high GSH metabolism group, whereas MXI1, THAP11, STAT4, and TBX21 were more active in the low GSH metabolism group (**Figure 4F**). Notably, MYC is a well-known regulator of metabolic processes, and MXI1 acts as its negative regulator.

Furthermore, a focused analysis of 12 immune-related transcription factors revealed significant activity differences between metabolic states (**Figure 4G**). STAT1 and RUNX1 exhibited enhanced activity in the high GSH metabolism group, both of which play roles in antitumor immune regulation, whereas key regulators such as NFATC1, TBX21, IRF4, EOMES, ZEB2, and GATA3 were upregulated in the low GSH metabolism group (**Figure 4G**). This pattern suggests that glutathione metabolism may modulate the tumor microenvironment’s immune response by regulating these immune-related transcription factors.

### 3.4 Remodeling of the intercellular communication network

**Figure 5:**
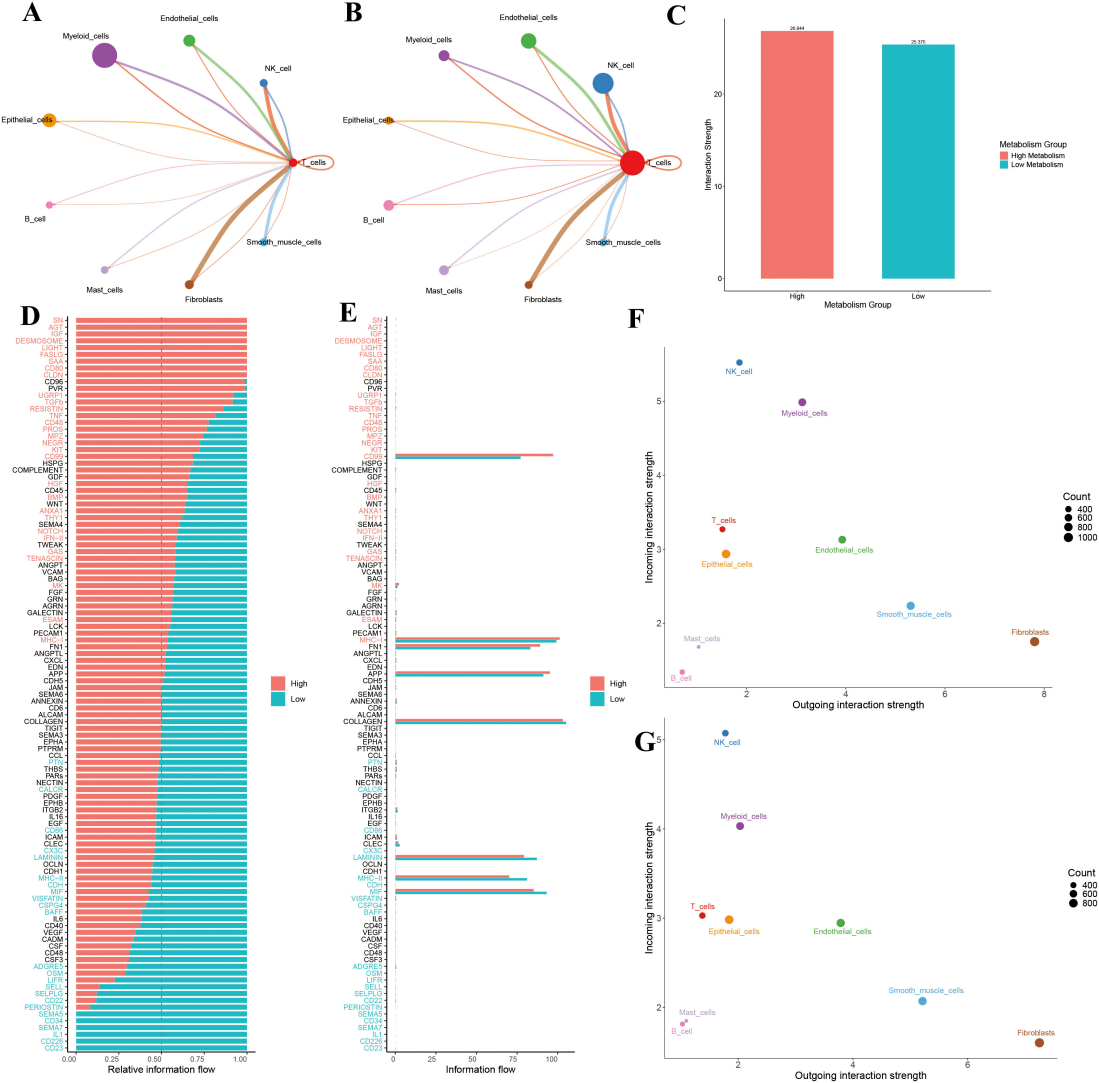
**(A)** T cell communication in the high-metabolism group. **(B)** T cell communication in the low-metabolism group. **(C)** Comparison of overall interaction strength between high and low metabolism groups. **(D)** Comparison of relative information flow between high and low metabolism groups. **(E)** Comparison of absolute information flow between high and low metabolism groups. **(F)** Scatter plot analysis of cellular communication patterns in the high-metabolism group. **(G)** Scatter plot analysis of cellular communication patterns in the low-metabolism group.

To visualize T cell communication within the LUAD microenvironment, we constructed a T cell–centered interaction network, which revealed communication differences between high and low GSH metabolism groups (**Figures 5A, B**). Comparative analysis showed that the high GSH metabolism group had a significantly higher average communication strength (26.844) compared to the low GSH metabolism group (25.375) (**Figure 5C**), suggesting that elevated glutathione metabolism may remodel the LUAD microenvironment by enhancing intercellular communication. Moreover, systematic analysis of signaling pathway activities between groups indicated that shifts in metabolic status were closely linked to the activation of specific pathways (**Figures 5D, E**). In the high GSH metabolism group, several key immune-regulatory pathways were markedly enhanced, including the MHC-I antigen presentation pathway, cytokine signaling pathways (IFN-II, TNF, TGFβ), costimulatory molecule pathway (CD80), and the cell death pathway (FASLG). Additionally, growth factor signaling (IGF, HGF, BMP) and cell adhesion-related pathways (DESMOSOME, CLDN, ESAM) exhibited higher activity in the high GSH metabolism group. In contrast, the low GSH metabolism group specifically activated another set of immune-regulatory pathways, including MHC-II, CD86, chemokine (CX3C), and adhesion molecules (SELL, SELPLG) (**Figures 5D, E**). These differential pathway activities imply that distinct metabolic states may influence the tumor microenvironment’s immune status by modulating different intercellular communication networks. Finally, analysis of signal input and output intensities across cell types (**Figures 5F, G**) revealed that T cells in the high GSH metabolism group exhibited slightly higher signal output, while signal input remained relatively stable between groups. Moreover, NK cells and myeloid cells in the high GSH metabolism group demonstrated comparatively balanced and higher input-output intensities than those in the low GSH metabolism group.

### 3.5 Analysis of CD8+ T cell subpopulations

**Figure 6:**
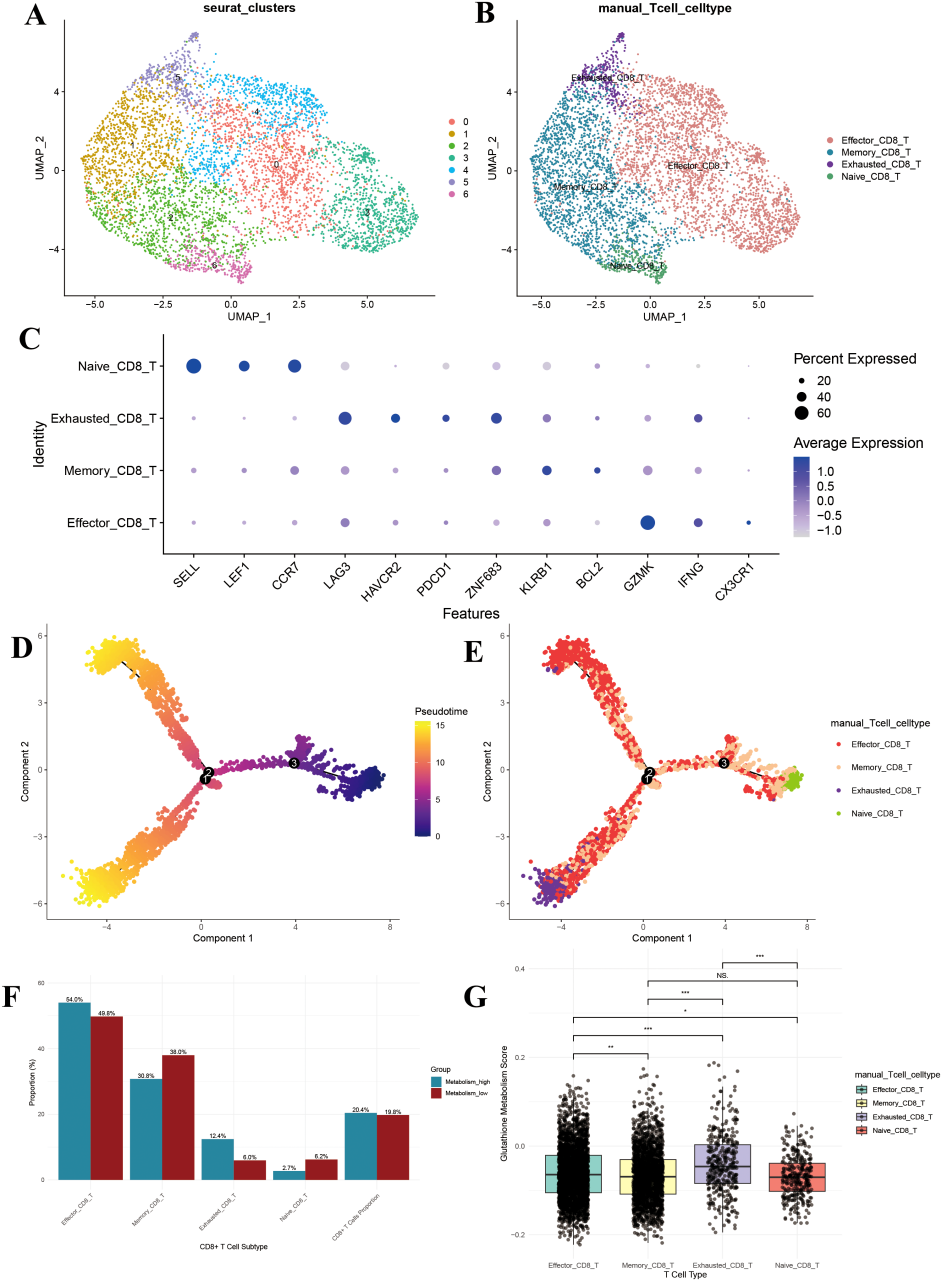
CD8+ T cell subpopulation analysis. **(A)** UMAP of the 7 CD8+ T cell clusters; **(B)** UMAP after CD8+ T cell annotation; **(C)** Dotplot of the marker gene for each CD8+ T cell type; **(D)** Monocle-based trajectory inference analysis colored by pseudotime; **(E)** Monocle-based trajectory inference analysis colored by CD8+ T cell type; **(F)** Bar plot of cell proportions in CD8+ T cells; **(G)** Box plot of glutathione metabolism levels in CD8+ T cell subpopulations. UMAP, uniform manifold approximation and projection. *P<0.05, **P<0.01, and ***P<0.001.

Through re-clustering and dimensionality reduction of CD8+ T cells, we identified seven distinct clusters (**Figure 6A**). Based on marker gene expression, these clusters were manually annotated into four major subpopulations: effector, memory, naïve, and exhausted CD8+ T cells (**Figure 6B**). Dot plot analysis revealed distinct gene expression profiles among these groups: naïve CD8+ T cells exhibited high expression of SELL, LEF1, and CCR7; exhausted CD8+ T cells specifically expressed LAG3, HAVCR2, and PDCD1; memory CD8+ T cells were enriched in ZNF683, KLRB1, and BCL2; and effector CD8+ T cells showed elevated levels of GZMK, IFNG, and CX3CR1 (**Figure 6C**). These patterns confirm the accuracy of our CD8+ T cell subpopulation annotations.

### 3.6 Developmental trajectory analysis of CD8+ T cells

Pseudotime analysis demonstrated that pseudotime commenced at zero and gradually increased along two primary branches (pseudotime > 10) (**Figure 6D**). The distribution of cell subpopulations along these trajectories revealed that naïve CD8+ T cells were predominantly located at the branch origins, subsequently differentiating into memory and effector CD8+ T cells, while exhausted CD8+ T cells mainly appeared at the terminal ends (**Figure 6E**). This spatial distribution aligns with the established differentiation patterns of CD8+ T cells, further validating our subpopulation annotations.

### 3.7 Increased proportion of exhausted CD8+ T cells in the high-metabolism group

To assess the impact of glutathione metabolism on CD8+ T cell subpopulation distribution, we compared the proportions of each subpopulation between the high and low GSH metabolism groups. The high GSH metabolism group exhibited a significantly higher proportion of effector CD8+ T cells (54.0% vs. 49.8%), while memory CD8+ T cells were less prevalent (30.8% vs. 38.0%) (**Figure 6F**). Notably, exhausted CD8+ T cells accounted for a substantially larger fraction in the high GSH metabolism group (12.4% vs. 6.0%), and naïve CD8+ T cells were less abundant (2.7% vs. 6.2%) (**Figure 6F**). Additionally, CD8+ T cells constituted a slightly higher percentage of total T cells in the high GSH metabolism group (20.4% vs. 19.8%) (**Figure 6F**). Further analysis of glutathione metabolism scores revealed that exhausted CD8+ T cells exhibited the highest metabolic activity, significantly surpassing that of the other subpopulations (P < 0.01) (**Figure 6G**).

### 3.8 Differential gene function enrichment analysis

Among the 12 core glutathione metabolism genes, all exhibited a significant positive correlation with the GSH score (**Supplementary Figure 1**). This confirms that our scoring system accurately reflects the transcriptional activity of the glutathione metabolism pathway. Analysis of central carbon metabolism revealed that all four carbon metabolic pathways exhibited higher activity in the high GSH metabolism group (**Supplementary Figure 2**).

**Figure 7:**
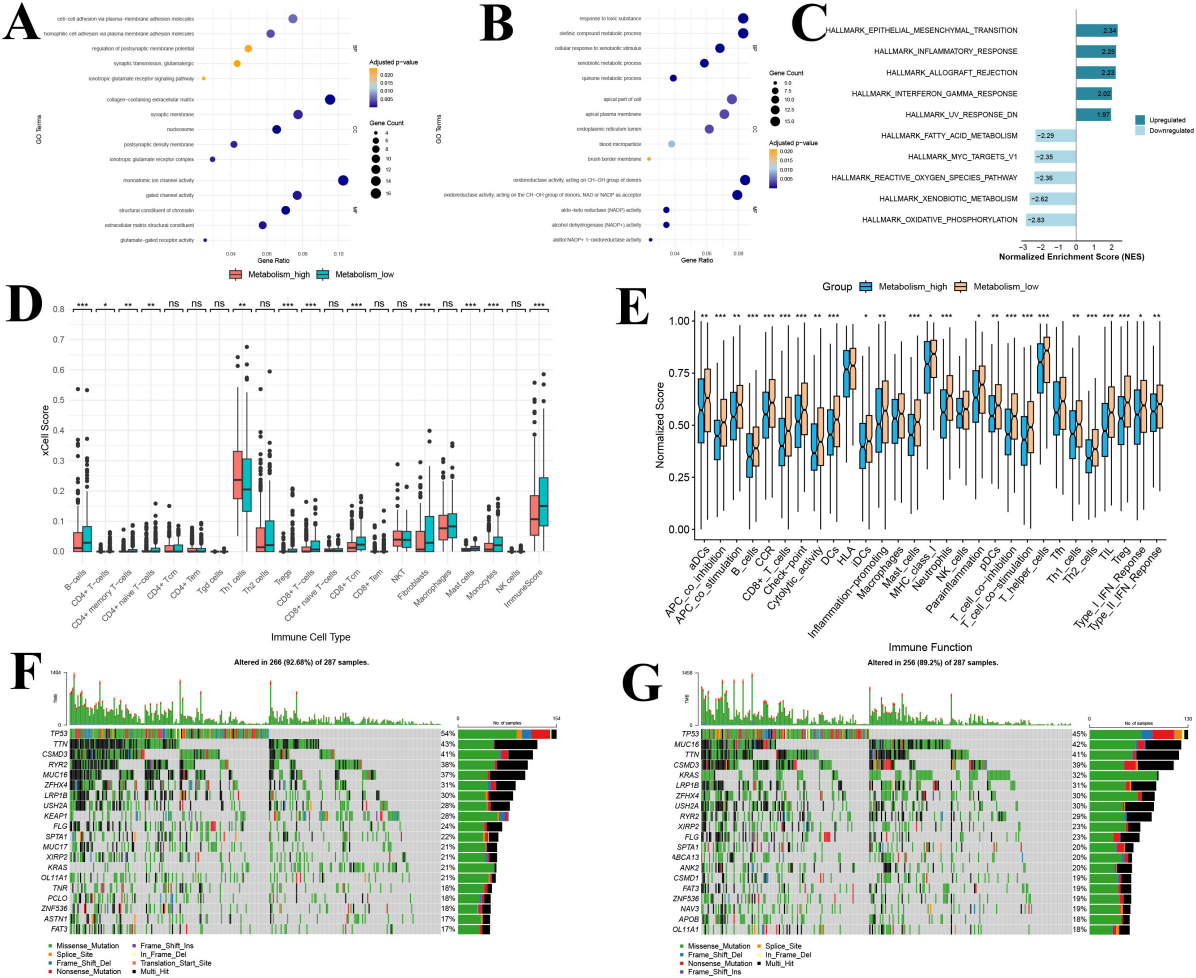
Functional enrichment, immune infiltration, and immune function analysis of the glutathione metabolism group in bulk transcriptomic data **(A)** GO analysis of upregulated DEGs; **(B)** GO analysis of downregulated DEGs; **(C)** Gene Set Enrichment Analysis of DEGs; **(D)** Immune infiltration analysis; **(E)** Immune functional analysis; **(F)** Waterfall plot of the top 20 most frequently mutated genes in the high GSH metabolism group; **(G)** Waterfall plot of the top 20 most frequently mutated genes in the low GSH metabolism group. GO, Gene Ontology; DEGs, differentially expressed genes; BP, Biological process; CC, Cellular component; MF, Molecular function; GSEA, Gene Set Enrichment Analysis. *P<0.05, **P<0.01, and ***P<0.001.

By comparing transcriptomic differences between the high and low GSH metabolism groups, 510 significantly differentially expressed genes were identified. Among these, 269 were upregulated while 241 were downregulated. Based on these genes, GO enrichment analyses were conducted separately on the upregulated genes from each group, revealing distinct functional patterns (**Figures 7A, B**). In the high GSH metabolism group, upregulated genes were predominantly enriched in pathways related to neuronal signal transduction and cell adhesion. Specifically, biological processes such as the ionotropic glutamate receptor signaling pathway and glutamatergic synaptic transmission were most prominent, indicating activation of neuro-like signaling under high glutathione metabolism. Cellular component enrichment included synaptic structures (e.g., ionotropic glutamate receptor complexes, postsynaptic density membranes) and extracellular matrix components. And molecular functions were mainly associated with receptor and channel activities, including glutamate-gated receptor and ion channel functions (**Figure 7A**). In contrast, enrichment analysis of genes upregulated in the low GSH metabolism group revealed functions related to both metabolism and detoxification. The Biological Process category included quinone metabolism, xenobiotic metabolism and response to toxic substances. Cellular Component terms were enriched for brush border membrane, endoplasmic reticulum lumen and apical cell structures. Molecular Function was dominated by various oxidoreductase activities, notably NADP−dependent dehydrogenase and reductase activities (**Figure 7B**).

GSEA further elucidated key signaling pathway differences between the high and low GSH metabolism groups (**Figure 7C**). In the high GSH metabolism group, five significantly upregulated Hallmark pathways were predominantly associated with tumor invasion and metastasis (epithelial–mesenchymal transition) and immune-inflammatory responses (inflammatory response, allograft rejection, and interferon-γ response). These findings suggest that lung adenocarcinoma with active glutathione metabolism may exhibit both enhanced invasiveness and a distinct immune microenvironment. Conversely, the five pathways enriched in the low GSH metabolism group were mainly related to fundamental metabolic functions such as energy metabolism (oxidative phosphorylation), xenobiotic processing, and oxidative stress response (reactive oxygen species metabolism). Notably, the negative enrichment of the reactive oxygen species metabolism pathway is closely tied to the role of glutathione as a primary antioxidant, implying that elevated glutathione metabolism may partially substitute for reactive oxygen species clearance mechanisms.

### 3.9 Immune infiltration, immune function and mutation analysis based on metabolic grouping

Using the xCell algorithm, we compared immune cell infiltration in the TCGA lung adenocarcinoma cohort between the high and low GSH metabolism groups (**Figure 7D**). Among the 20 immune cell types evaluated, statistically significant differences between the groups were observed in 11 cell types and in the overall immune score (P < 0.05). Notably, compared with the low GSH metabolism group, tumor tissues from patients in the high GSH metabolism group exhibited significantly lower levels of CD8+ T cells, CD8+ T central memory cells, and overall immune scores (P < 0.05) (**Figure 7D**). This suggests that elevated glutathione metabolism may be associated with reduced infiltration of various immune cells, particularly T cells, in the tumor microenvironment of lung adenocarcinoma patients.

Furthermore, ssGSEA analysis demonstrated that differences in glutathione metabolism exert a broad impact on immune function in lung adenocarcinoma (**Figure 7E**). Among the 29 immune functions evaluated, 25 functions showed significant differences between the two groups (P < 0.05). Compared to the low GSH metabolism group, patients in the high GSH metabolism group exhibited generally suppressed immune functions, as evidenced by marked downregulation of antigen presentation-related functions (including dendritic cell activities encompassing DCs, aDCs, iDCs, and pDCs, as well as MHC-I molecule expression); reduced T cell activation and function, reflected by lower expression of T cell costimulatory molecules and diminished T helper cell (Th1 and Th2) activities; weakened effector immune responses involving decreased cytotoxic activity, B cell function, and inflammatory response; and significantly attenuated type I and type II interferon responses (**Figure 7E**). Notably, CD8+ T cell function was lower in the high GSH metabolism group than in the low GSH metabolism group, which aligns with our conclusions. Additionally, the expression of immune checkpoint-related genes was significantly reduced in the high GSH metabolism group, suggesting that glutathione metabolism may modulate tumor immune responses by affecting immune checkpoint molecule expression.

Somatic mutation analysis revealed distinct mutational patterns between the high GSH metabolism group and the low GSH metabolism group (**Figures 7F, G**). In the high GSH metabolism cohort (n=287), TP53 was the most frequently mutated gene (54%), followed by TTN (43%), CSMD3 (41%), and RYR2 (38%) (**Figure 7F**). Notably, KEAP1 mutations were observed in 28% of high GSH metabolism tumors (**Figure 7F**). In contrast, in the low GSH metabolism cohort (n=287), TP53 remained the most commonly mutated gene (45%) (**Figure 7F**). However, KRAS mutations were significantly more prevalent in the low GSH metabolism group compared to the high GSH group (32% vs. 21%) (**Figure 7G**). Additionally, MUC16 mutations were more frequent in the low-GSH metabolism cohort (42% vs. 37%) (**Figure 7G**).

### 3.10 WGCNA analysis for selection of intersection genes

**Figure 8:**
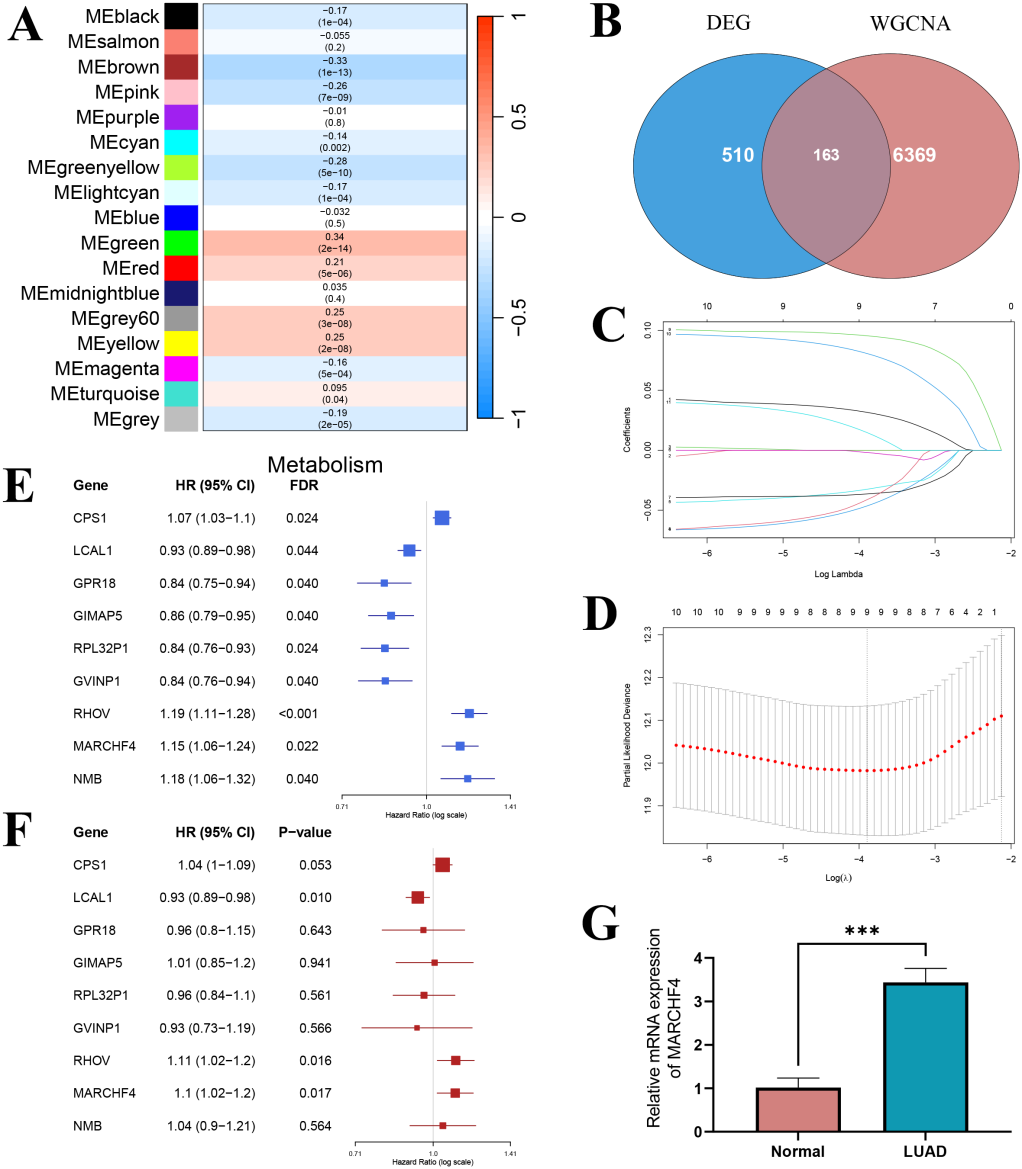
Screening of prognostic genes. **(A)** Heatmap of the correlation between module eigengenes and glutathione metabolism; **(B)** Venn diagram showing the intersection between WGCNA hub genes and differential genes; **(C)** LASSO regression coefficient curve; **(D)** LASSO regression results; **(E)** Univariate COX regression forest plot; **(F)** Multivariate COX regression forest plot. **(G)** The mRNA expression of MARCHF4 in normal and lung adenocarcinoma cell lines. ***P<0.001.

Using WGCNA, 17 co-expression modules were identified, of which 5 modules were significantly correlated with glutathione metabolism levels (|r| > 0.25, P < 0.05): the green module (r = 0.34, P = 2e-14) and the yellow module (r = 0.25, P = 2e-08) showed positive correlations, whereas the brown (r = -0.33, P = 1e-13), pink (r = -0.26, P = 7e-09), and green-yellow (r = -0.28, P = 5e-10) modules exhibited negative correlations (**Figure 8A**). Among these, the brown module contained 1,933 genes (84 overlapping with the differentially expressed genes), the pink module contained 883 genes (14 overlapping), the green-yellow module contained 884 genes (38 overlapping), the green module contained 1,162 genes (6 overlapping), and the yellow module contained 1,507 genes (21 overlapping). By intersecting the 6,369 genes from these five modules with the 510 differentially expressed genes, we identified 163 candidate core module genes (**Figure 8B**). These genes are both central in the co−expression network and significantly differentially expressed between the high and low glutathione metabolism groups.

### 3.11 LASSO-Cox regression for prognostic gene selection

Univariate Cox regression analysis was performed on the 163 candidate core module genes to assess their association with overall survival in lung adenocarcinoma patients. After FDR correction, 14 genes were found to be significantly associated with overall survival (P < 0.05). To mitigate multicollinearity and build a more parsimonious prognostic model, LASSO regression analysis was conducted on these 14 genes. The LASSO coefficient path diagram showed that, as the penalty parameter λ increased, most gene coefficients gradually shrank to zero (**Figure 8C**). Ten-fold cross-validation determined the optimal λ value, and at the minimum error, 9 genes with non-zero coefficients were selected as candidate genes for the prognostic model (**Figure 8D**). Univariate analysis revealed that high expression of CPS1, RHOV, MARCHF4, and NMB were significantly associated with poorer prognosis (HR > 1), whereas high expression of LCAL1, GPR18, GIMAP5, RPL32P1, and GVINP1 were significantly associated with better prognosis (HR < 1) (**Figure 8E**). When these 9 LASSO-selected genes were incorporated into a multivariate Cox regression model, LCAL1, RHOV, and MARCHF4 remained significantly associated with overall survival after controlling for the influence of other genes (P < 0.05) (**Figure 8F**). Specifically, high LCAL1 expression acted as a protective factor, while high expression of RHOV and MARCHF4 conferred an increased risk. These three genes were thus determined as the final markers for constructing the prognostic gene risk score model. Given that LCAL1 and RHOV have been previously reported as prognostic genes, we validated the novel prognostic gene MARCHF4 via qRT-PCR, demonstrating that its expression was significantly upregulated in lung adenocarcinoma cells compared to normal lung epithelial cells (**Figure 8G**).

### 3.12 Construction of the metabolic risk score and grouping

Based on the expression levels of the 3 prognostic genes in the TCGA lung adenocarcinoma cohort, a gene risk score model was established using the following formula:


$$\begin{array}{c} \text{Gene risk score }=\;(–0.13490\;\times \text{ LCAL}1\text{ expression})\;+\;(0.33335\;\\ \times \text{ RHOV expression})\;+\;(0.23015\\ \;\times \text{ MARCHF}4\text{ expression}). \end{array}$$

**Figure 9:**
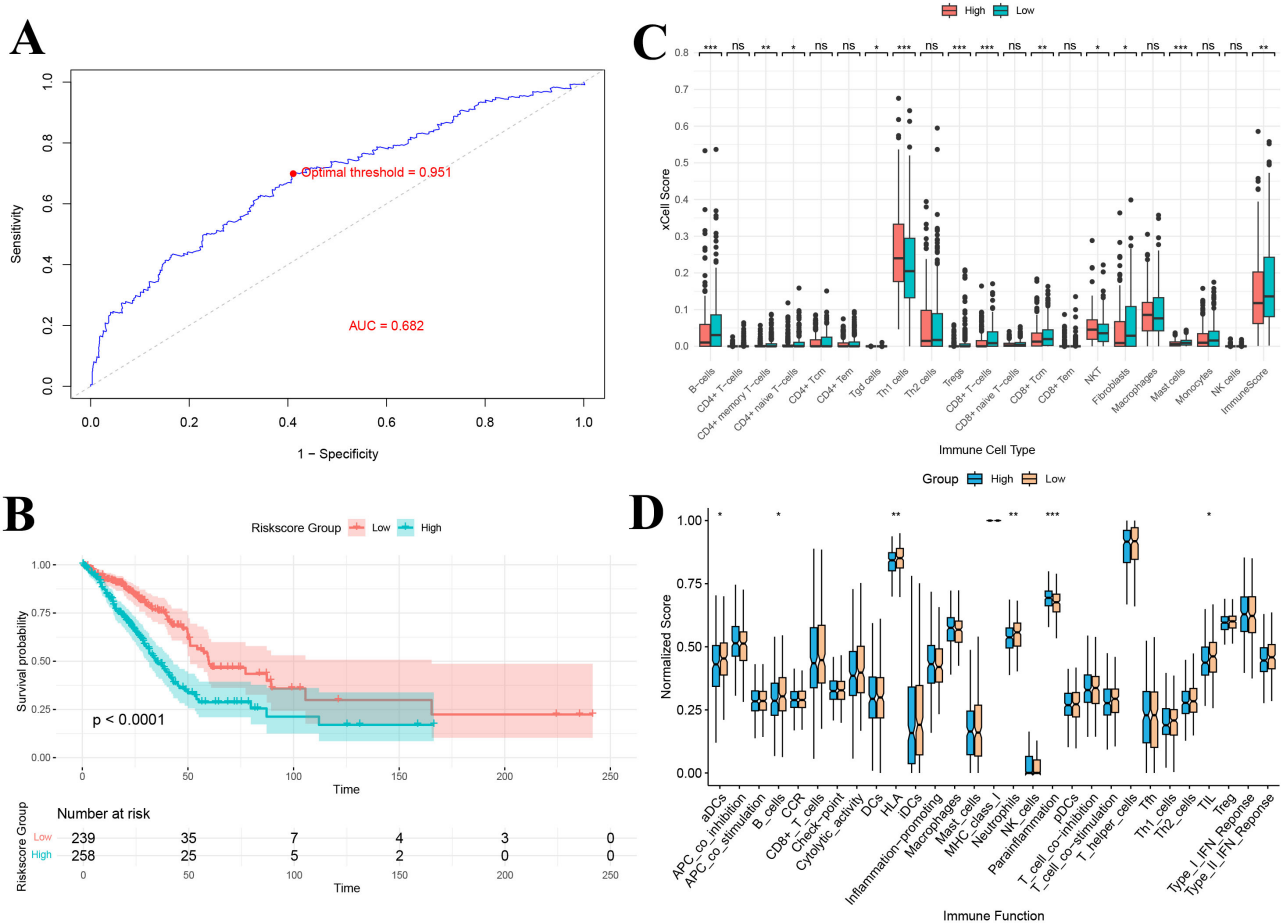
**(A)** ROC-based criteria for grouping by gene score; **(B)** Kaplan–Meier curve of gene score grouping; **(C)** Immune infiltration analysis of gene score grouping; **(D)** Immune function analysis of gene score grouping. *P<0.05, **P<0.01, and ***P<0.001.

The optimal cutoff value (0.951), determined by the maximum area under the ROC curve (AUC = 0.682) for 36-month survival prediction, was used to stratify patients into a high gene score group (n = 258) and a low gene score group (n = 239) (**Figure 9A**). Kaplan-Meier survival analysis showed a significant difference in overall survival between the two groups, with patients in the high gene score group exhibiting markedly lower survival rates compared to those in the low gene score group (P < 0.0001) (**Figure 9B**).

### 3.13 Immune infiltration and immune function analysis based on gene score grouping

xCell immune infiltration analysis revealed significant differences in the levels of 11 immune cell types between the high and low gene score groups (**Figure 9C**). Notably, the overall immune score was significantly lower in the high gene score group, indicating reduced immune cell infiltration (P < 0.01). Additionally, immune function analysis demonstrated significant differences in 6 immune-related functions between the groups, including antigen-presenting cell (aDCs) activity, B cell function, HLA expression, neutrophil activity, anti-inflammatory response, and tumor-infiltrating lymphocytes (TILs) (**Figure 9D**). The downregulation of antigen presentation-related functions (e.g., HLA and aDCs) suggests that high gene score group patients may have impairments in antigen recognition and presentation, potentially affecting subsequent T cell activation and effector responses.

### 3.14 Immune therapy and drug sensitivity prediction

**Figure 10:**
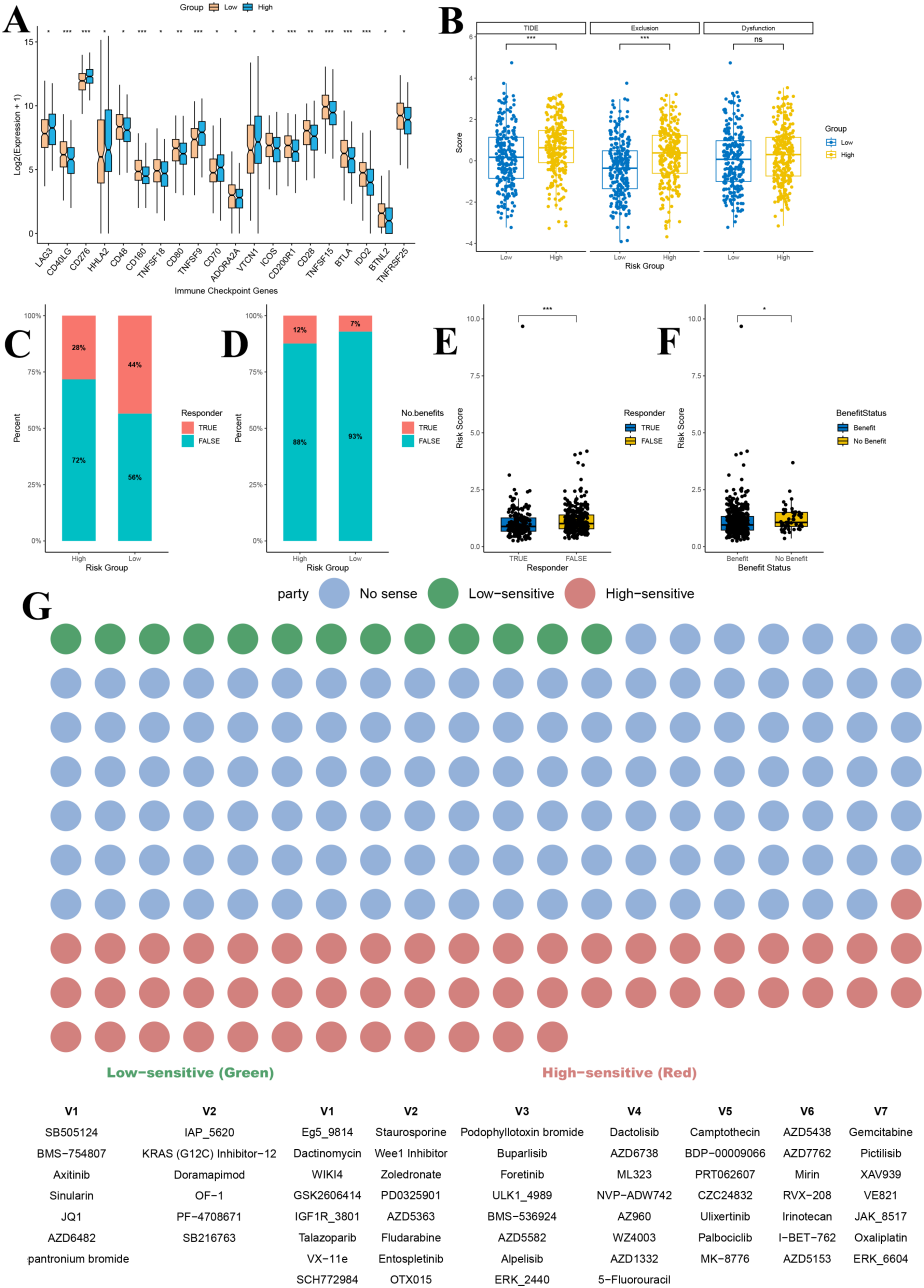
Immunotherapy and drug sensitivity prediction analysis based on gene risk score grouping. **(A)** Immune checkpoint analysis of gene score grouping; **(B)** TIDE analysis of gene score grouping; **(C)** Predicted proportion of immunotherapy responders in high and low gene score groups; **(D)** Predicted proportion of immunotherapy benefit in high and low gene score groups; **(E)** Comparison of risk scores between immunotherapy responders and non-responders; **(F)** Distribution of risk scores among patients with different treatment benefit statuses; **(G)** Drug sensitivity prediction for patients in high and low gene score groups. TIDE, Tumor Immune Dysfunction and Exclusion. *P<0.05, **P<0.01, and ***P<0.001.

Analysis of immune checkpoint genes in the TCGA lung adenocarcinoma dataset successfully identified 20 immune checkpoint genes that were significantly differentially expressed between the high and low gene score groups (**Figure 10A**). These genes exhibited distinct expression patterns between the two groups. In particular, LAG3, CD276, HHLA2, TNFSF9, CD70 and VTCN1 were significantly upregulated in the high gene score group compared to the low gene score group (**Figure 10A**). Notably, the remaining checkpoints, including CD40LG, CD48, CD160, TNFSF18, CD80, ADORA2A, ICOS, CD200R1, CD28, TNFSF15, BTLA, IDO2, BTNL2 and TNFRSF25, were markedly downregulated in the high gene risk group (P < 0.05) (**Figure 10A**).

Furthermore, comparison of TIDE scores between the high and low gene score groups revealed that patients in the high gene score group had significantly higher overall TIDE scores, suggesting a poorer predicted response to immunotherapy (P < 0.001) (**Figure 10B**). Among the two TIDE components, the immune exclusion score was significantly higher in the high gene score group (P < 0.001), indicating stronger suppression of immune cell infiltration in these tumors, while the T cell dysfunction score did not differ significantly between the groups (P > 0.05) (**Figure 10B**).

To further assess the predictive value of our risk model for immunotherapy efficacy, we analyzed the response rates to immune checkpoint inhibitors. The proportion of responders in the low gene score group (44%) was substantially higher than in the high gene score group (28%) (**Figure 10C**). Similarly, the percentage of patients deriving clinical benefit from immunotherapy was higher in the low gene score group (93%) compared to the high gene score group (88%) (**Figure 10D**). Additionally, risk scores were significantly lower in immunotherapy responders than in non-responders (P < 0.001) and in patients predicted to benefit from treatment compared with those predicted not to benefit (P < 0.05) (**Figures 10E, F**).

Drug sensitivity analysis further demonstrated that patients in the high and low gene score groups exhibited significant differences in sensitivity to various anticancer agents (**Figure 10G**). Across the entire drug dataset, 13 drugs showed higher sensitivity in the low gene score group, while 53 drugs exhibited higher sensitivity in the high gene score group (P < 0.05) (**Figure 10G**). Among LUAD–related therapies, the KRAS (G12C) inhibitor-12 showed markedly higher sensitivity in the low gene score group, suggesting that these patients might benefit more from KRAS-targeted therapy (**Figure 10G**). Conversely, in the high gene score group, Gemcitabine, PD0325901 (a MEK inhibitor), Ulixertinib (an ERK inhibitor), and Palbociclib (a CDK4/6 inhibitor) displayed greater sensitivity, indicating a potentially better therapeutic response to these agents (**Figure 10G**).

### 3.15 Construction of the prognostic model

**Figure 11:**
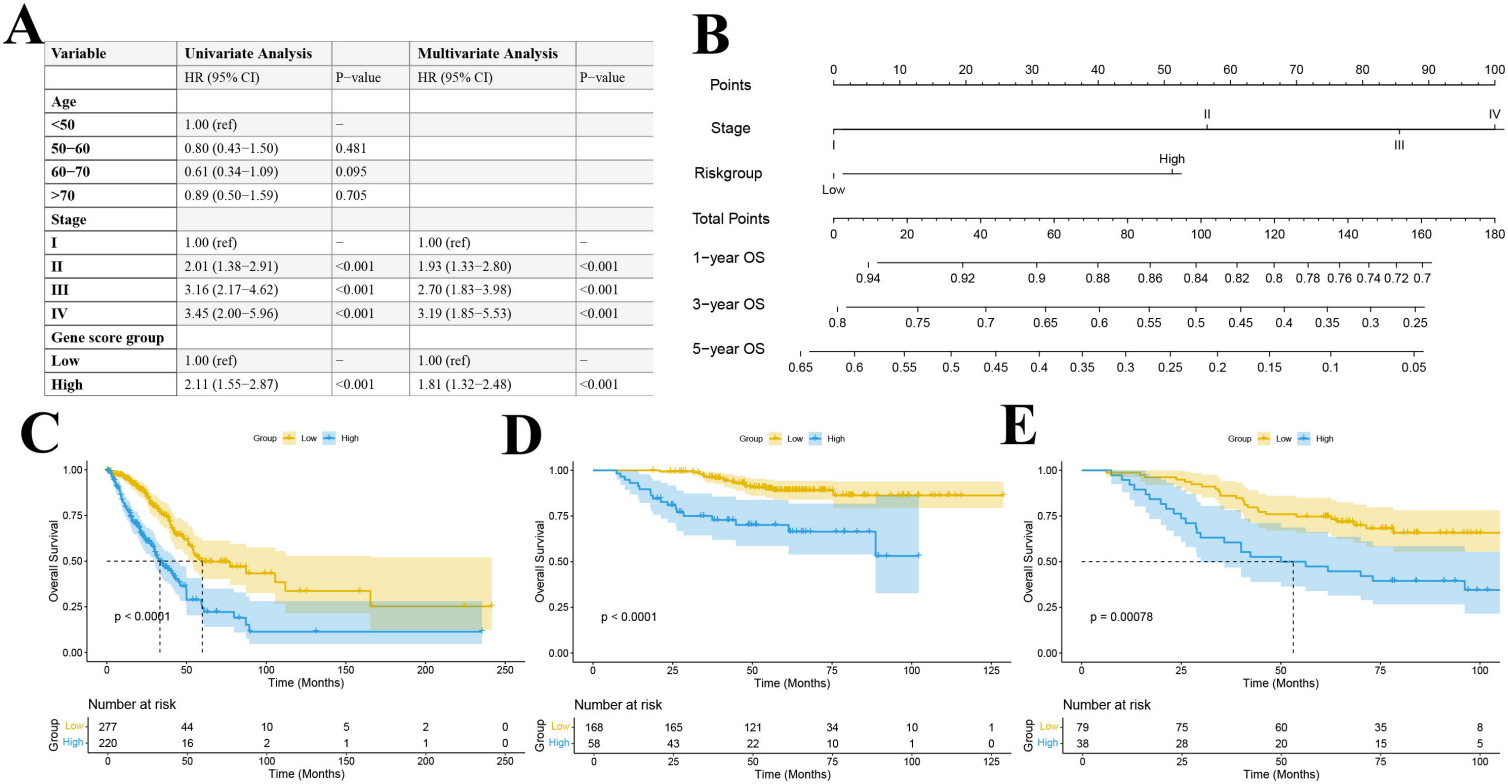
**(A)** Univariate and multivariate Cox regression table; **(B)** Construction of a prognostic nomogram; Kaplan–Meier survival curve for the TCGA training set **(C)** and GEO datasets GSE31210 **(D)** and GSE13213 **(E)**.

We used the TCGA lung adenocarcinoma dataset (n = 497) as the training set, and the GEO datasets GSE31210 (n = 226) and GSE13213 (n = 117) as independent validation cohorts. The baseline characteristics of the training and validation cohorts are presented in **Supplementary Table 4**. A univariate cox regression analysis revealed that tumor stage and the gene−based risk score were significantly associated with overall survival (**Figure 11A**). Importantly, differences in survival across age strata were not statistically significant. Multivariate cox regression analysis further confirmed the independent prognostic value of tumor stage and risk score (**Figure 11A**).

By integrating these independent prognostic factors, we constructed a comprehensive prognostic nomogram that incorporated both clinical pathological features and gene expression profiles (**Figure 11B**). This nomogram, which includes tumor stage and risk score as independent predictors, was designed to estimate 1-, 3-, and 5-year overall survival probabilities. Each variable was assigned a score based on its Cox regression coefficient, and the total score corresponded to the predicted survival probability.

### 3.16 Validation of the prognostic model

**Figure 12:**
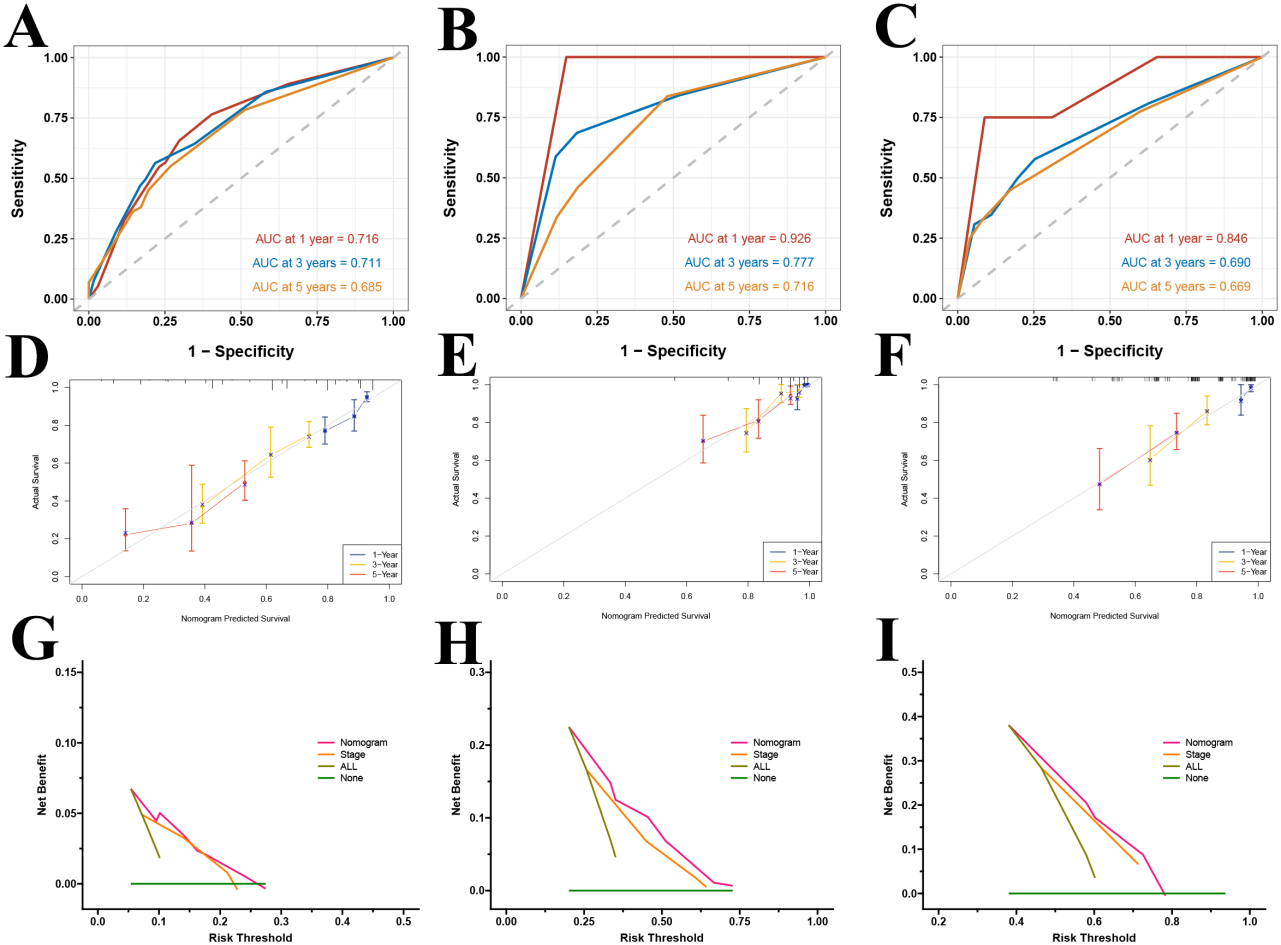
Validation of the prognostic model. ROC curves for the TCGA training set **(A)** and GEO datasets GSE31210 **(B)** and GSE13213 **(C)**; calibration curves for the TCGA training set **(D)** and GEO datasets GSE31210 **(E)** and GSE13213 **(F)**; DCA curves at 1-year **(G)**, 3-year **(H)**, and 5-year **(I)** for the TCGA dataset.

Patients were stratified into high- and low-risk groups based on the median predicted total score. Kaplan-Meier survival analyses in both the training and validation cohorts demonstrated a significant difference in overall survival between the risk groups, with the low-risk group exhibiting significantly better survival outcomes (P < 0.001) (**Figures 11C–E**). ROC curve analysis revealed that our multivariable prognostic model achieved good predictive performance in the TCGA-LUAD training set, with 1-, 3-, and 5-year AUC values of 0.716, 0.711, and 0.685, respectively (**Figure 12A**). In two independent validation cohorts, the model also demonstrated stable predictive accuracy. In the GSE13213 cohort, the area under the ROC curve at 1-, 3-, and 5-year was 0.926, 0.777 and 0.716, respectively (**Figure 12B**). In the GSE13213 cohort, these values were 0.846, 0.690 and 0.669 (**Figure 12C**). These findings indicate that the prognostic model has robust predictive capability across different populations and datasets. Calibration curves further demonstrated a high degree of concordance between predicted and observed survival probabilities in all three cohorts (**Figures 12D–F**). Decision curve analysis showed that the nomogram model integrating the gene risk score with clinical factors provided greater clinical utility compared to models based solely on tumor stage (**Figures 12G–I**). Collectively, these validation results support the accuracy and potential clinical applicability of the prognostic model.

## 4 Discussion

This study integrates genetic causal analysis, single-cell RNA sequencing, and bulk transcriptome analysis to elucidate the metabolic and immunological associations of glutathione in lung adenocarcinoma, particularly its effects on T cells within the tumor microenvironment. This discovery provides a novel perspective on the pathogenesis of lung adenocarcinoma. Furthermore, the prognostic risk model based on genes related to differences in glutathione metabolism offers robust evidence for predicting immune treatment responses, drug sensitivity, and survival outcomes, thereby laying the groundwork for personalized therapeutic strategies.

Our genetic causal analysis demonstrated that elevated levels of the glutathione metabolic intermediate 5-oxoproline are associated with an increased risk of lung adenocarcinoma. This finding aligns with previous research highlighting the critical role of glutathione metabolism in cancer development (7, 10). Elevated glutathione levels help maintain tumor cell homeostasis, thus promoting cell proliferation and survival. Notably, mediation analysis identified that CD28^–^CD25^++^ CD8^+^ T cells serve as mediators between 5-oxoproline and lung adenocarcinoma. These observations suggest that glutathione metabolism may partially elevate tumor risk by modulating the number and function of antitumor immune cells (4, 15–17). This is the first study to genetically implicate immune cells as mediators between lung adenocarcinoma and the glutathione metabolite 5-oxoproline, thus offering fresh insights into the interplay between glutathione metabolism and tumor immunity.

Furthermore, the genetic causal analysis results in this study were not adjusted for FDR, a practice observed in previous investigations (51–55). During the selection of instrumental variables, strict p-value thresholds and independence screening were employed to minimize false positives. Although FDR correction is effective in controlling error rates in high-dimensional data, overly stringent adjustments may obscure potential associations when the number of hypotheses tested is limited. Recent studies have underscored inherent challenges in genetic causal inference, specifically weak instrument bias, population stratification and selection bias (56–60). To mitigate these issues, we utilized GWAS summary statistics from multiple ancestral populations. Additionally, we performed extensive sensitivity analyses including heterogeneity tests, pleiotropy assessments and the MR PRESSO procedure. These analyses revealed no significant heterogeneity or horizontal pleiotropy and thus reinforced the robustness of our findings. We also conducted bidirectional analyses to rule out reverse causation. These analyses detected no evidence of bias. It is important to emphasize that this genetic causal analysis is exploratory in nature, aiming to provide new insights into the relationship between glutathione metabolism and immunity. Future studies should incorporate more diverse populations and additional statistical methods to further validate these findings.

Previous studies have used GWAS data to examine the relationship between 731 immune cell phenotypes and lung cancer via genetic causal analysis (51, 54, 61, 62). However, the mediating role of metabolites was not explored. Moreover, our study utilized lung adenocarcinoma data from the Finngen R11 dataset, which is based on the Finnish Biobank and includes genomic and health data from approximately 500,000 participants. The unique genetic background and environmental factors of the Finnish population enable our study to offer genetic association insights that differ from those obtained in other populations.

Subsequent analysis using single-cell sequencing data further investigated the impact of glutathione metabolic reprogramming on the tumor microenvironment. Analysis of transcription factor activities revealed significant effects on immune regulation. For instance, MYC, which is a well-known regulator of metabolism that controls glycolysis, glutamine transport and metabolism, lipid metabolism, and mitochondrial biogenesis, was more active in the high GSH metabolism group, suggesting that elevated GSH levels may drive tumor cell metabolic demands and rapid proliferation through MYC activation (63). Additionally, NFE2L2 (also known as NRF2), a key regulator of oxidative stress, appeared activated in high-GSH cells due to increased redox pressure (64). Its activation promotes the expression of genes involved in GSH synthesis and GPX4, thereby enhancing the antioxidant capacity of cells and establishing a positive feedback loop that maintains redox homeostasis in tumor cells. Conversely, in the low GSH metabolism group, transcription factors such as MXI1 (a MYC antagonist), THAP11, STAT4, and TBX21 exhibited significantly enhanced activity. The increased activity of MXI1 could attenuate MYC-mediated transcription, thereby mitigating MYC-driven metabolic regulation and limiting abnormal tumor cell proliferation (65). Moreover, enhanced activities of STAT4 and TBX21, both critical for antitumor immunity, with STAT4 promoting IFN-γ production during Th1 differentiation and T-bet facilitating NK cell maturation and cytotoxic function, further underline the differential immune responses between the metabolic groups (66, 67).

Further examination of 12 transcription factors closely associated with immune function demonstrated marked differences between the high and low GSH metabolism groups. In the high GSH metabolism group, STAT1 and RUNX1 were significantly activated. STAT1, a pivotal mediator of interferon (IFN) signaling, not only enhances antitumor immune responses but may also contribute to chronic inflammation and immune evasion when persistently activated (67). Similarly, increased RUNX1 activity can promote tumor cell proliferation, migration, and invasion, as well as modulate angiogenesis and the immune microenvironment, thereby accelerating malignant progression (68). The activation of these factors in the high GSH metabolism group may coincide with the initiation of immunosuppressive mechanisms, ultimately leading to a diminished overall antitumor immune effect. In contrast, in the low GSH metabolism group, key immune regulatory transcription factors, including NFATC1, TBX21, IRF4, EOMES, ZEB2, and GATA3, were markedly activated. These factors are essential for T cell activation, differentiation, and functional maintenance. For example, NFATC1 is central to T cell activation and cytokine expression, while IRF4, EOMES, and GATA3 play critical roles in T cell differentiation and sustaining immune responses (69–72). The heightened activity of these factors suggests that the low GSH metabolism group may exhibit a more robust antitumor immune response, thereby enhancing effector functions and promoting tumor clearance.

The cell–cell communication analysis revealed that variations in glutathione metabolic levels significantly affect intercellular signaling networks. Specifically, the average communication intensity in the high GSH metabolism group was markedly greater than in the low GSH metabolism group, and the two groups exhibited pronounced differences in signaling pathway activity and communication patterns. Notably, the MHC-I pathway was markedly enhanced in the high GSH metabolism group; conversely, the low GSH metabolism group exhibited specific activation of the MHC-II pathway. The observed differences in MHC-I and MHC-II expression patterns suggest that varying glutathione metabolic levels may modulate the function of antigen-presenting cells and, consequently, influence the type of adaptive immune response.

The study further analyzed CD8^+^ T cell subpopulations by clustering and annotating them, comparing differences between high and low GSH metabolism groups. Pseudotime analysis delineated the developmental trajectory of CD8^+^ T cells, thereby validating the accuracy of the subpopulation annotations. Exhausted CD8^+^ T cells predominantly occupied the terminal stages of this trajectory and exhibited significantly higher glutathione metabolic activity compared to other subgroups. Furthermore, the proportion of exhausted CD8^+^ T cells was significantly increased in the high GSH metabolism group, suggesting that an elevated glutathione metabolic state may drive CD8^+^ T cells toward an exhausted phenotype, thereby diminishing their antitumor efficacy.

The high GSH metabolism group demonstrated coordinated upregulation of glycolysis, the tricarboxylic acid cycle, the pentose phosphate pathway, and amino acid metabolism; together, these pathways support the tumor’s antioxidant capacity and rapid proliferation. Maintaining elevated GSH levels requires an abundant supply of carbon sources and reducing equivalents (73). Intermediates generated by glycolysis feed into the PPP, producing NADPH, which is used to reduce oxidized glutathione back to GSH (74). At the same time, glyceraldehyde-3-phosphate enters the serine-glycine pathway, supplying the essential precursor glycine for GSH synthesis and generating additional NADPH via one-carbon metabolism. Moreover, high-GSH tumors often exhibit active glutaminolysis and robust TCA cycle activity, providing both glutamate and ATP to support GSH biosynthesis and cell proliferation (73). This high-throughput metabolic state profoundly impacts the TME, particularly by promoting immunosuppression. On the one hand, tumor cells’ excessive consumption of glucose and other nutrients creates competitive nutrient deprivation, impairing the function of tumor-infiltrating lymphocytes (such as effector T cells) due to glucose scarcity (15). Studies have shown that high glucose uptake by tumors reduces T-cell mTOR activity and interferon-γ production, thereby weakening antitumor immune responses (15). On the other hand, the large amounts of lactate produced by accelerated glycolysis acidify the TME. Low pH and lactate together drive immunosuppression. Lactate accumulation promotes polarization of tumor-associated macrophages toward an M2 phenotype, leading them to secrete inhibitory cytokines (e.g., interleukin-10) and express PD-L1, which in turn suppresses T-cell function (75). In addition, an acidic, lactate-rich environment directly induces apoptosis and dysfunction in effector T cells and, via hypoxia-inducible factor 1α (HIF-1α), upregulates PD-L1 on tumor cells, further impairing CD8^+^ T-cell activity (75). High intracellular GSH itself has also been linked to immune-tolerant mechanisms. For example, Zhang et al. found that elevated GSH in the TME cooperates with immunosuppressive IgG4 antibodies to weaken antibody-dependent cellular cytotoxicity and other effector functions, driving macrophages and lymphocytes toward a tolerogenic state and thereby significantly promoting tumor immune evasion and growth (76). Likewise, “redox-high” tumors with high GSH and NRF2 activity often lack tissue-resident memory T cell infiltration and respond poorly to immunotherapy (77). In summary, high-GSH metabolism meets tumors’ antioxidant needs by enhancing central carbon flux but simultaneously creates a nutrient-deprived, acidic, immunosuppressive microenvironment that inhibits antitumor immune cell function and enables immune escape.

The functional enrichment analysis of bulk transcriptome data also provided supporting evidence. Gene enrichment in the high GSH metabolism group was associated with neural signaling pathways and cell adhesion functions, and the upregulation of the epithelial–mesenchymal transition (EMT) pathway indicated that glutathione metabolism may influence tumor invasion and metastasis by modulating neuro-like signaling and promoting EMT. Concurrently, enrichment of immune and inflammatory pathways such as inflammatory response, allograft rejection and interferon gamma response in the high GSH metabolism group underscores the close connection between glutathione metabolism and immune function. Conversely, the enrichment of metabolic pathways (oxidative phosphorylation, xenobiotic metabolism, and reactive oxygen species metabolism) in the low GSH metabolism group reflects an intrinsic link between glutathione metabolism and redox balance.

Integrated transcriptomic immune infiltration analysis revealed that elevated glutathione metabolism correlates with reduced infiltration of multiple immune cell populations. These findings align with recent studies on the interaction between tumor metabolism and the immune microenvironment (3, 78). Notably, the high GSH metabolism group exhibited lower immune scores and CD8+ T cell proportions compared to the low-metabolism group, suggesting that glutathione metabolism may regulate anti-tumor immune responses by influencing T cell differentiation and functional states. This observation was corroborated by both our single-cell sequencing data and genetic causal analysis. The single-cell sequencing results similarly demonstrated that high glutathione metabolism levels are associated with CD8+ T cell exhaustion. Correspondingly, genetic causal analysis identified CD28−CD25++ CD8+ T cells as playing a crucial intermediary role in the relationship between elevated levels of 5-oxoproline and increased lung adenocarcinoma risk.

Moreover, immune function analysis revealed that elevated glutathione metabolism correlated with a decline in multiple immune functions, including antigen presentation, T cell activation, effector immune responses, and cytokine signaling. Particularly, the marked suppression of the interferon signaling pathway may be a key mechanism underlying the weakened antitumor immune response, given that interferon-γ is essential for the cytotoxic functions of macrophages and T cells in tumor cell clearance (79).

Tumor mutation profiling has revealed that the high GSH metabolism group is often accompanied by driver mutations in key genes such as TP53 and KEAP1. In the context of TP53 mutation, p53 loses its ability to regulate cellular redox homeostasis; mutant p53 cannot perform its normal antioxidant functions, resulting in elevated intracellular ROS (80). To survive, these tumor cells tend to reprogram their metabolism to upregulate GSH synthesis, thereby neutralizing excess ROS and maintaining redox balance (81). Conversely, some studies have shown that accumulated mutant p53 can interact with NRF2 to repress SLC7A11 expression, weakening GSH synthesis and rendering these cells more susceptible to oxidative damage (82). In addition, inactivating mutations of KEAP1 relieve its suppression of NRF2, and persistently activated NRF2 upregulates a series of antioxidant and detoxification genes, including the glutamate–cysteine ligase subunits GCLC/GCLM and the cytosolic SLC7A11 (83–85). This confers on tumor cells an enhanced capacity for GSH production and bolstered antioxidant reserves. NRF2-mediated reprogramming of GSH metabolism not only helps tumors withstand oxidative stress but is also closely linked to immune evasion (77). Tumors with high NRF2 activity often exhibit reduced T-cell infiltration and an “immune-cold” phenotype (77). In addition, KEAP1 mutations impair PD-L1 degradation, leading to PD-L1 accumulation and suppression of antitumor immunity, thereby facilitating immune escape (86). In summary, mutations in TP53, KEAP1, and related pathways reprogram GSH metabolism and redox signaling to endow tumors with increased antioxidant capacity and, in concert, promote immune evasion.

In summary, our genetic data analysis established a causal link between increased levels of the glutathione metabolic intermediate 5-oxoproline and a heightened risk of lung adenocarcinoma, with CD28–CD25++ CD8^+^ T cells playing a crucial mediating role. These findings reveal a close association between aberrant glutathione metabolism and immunosuppression in lung adenocarcinoma. Subsequent single-cell sequencing and bulk transcriptome analyses demonstrated that glutathione metabolic reprogramming remodels the tumor immune microenvironment by modulating key transcription factor activities, altering cell–cell communication networks, and promoting CD8^+^ T cell exhaustion. Collectively, these discoveries provide new insights into the mechanisms underlying lung adenocarcinoma progression and offer a theoretical basis for precision therapies targeting glutathione metabolism in combination with immunotherapy.

This study employed WGCNA to select intersecting differentially expressed genes and combined Lasso-Cox regression to identify three independent prognostic markers: LCAL1, RHOV, and MARCHF4. LCAL1, a newly discovered long noncoding RNA associated with lung cancer, has not yet been extensively studied; our findings indicate that its high expression correlates with improved prognosis in lung adenocarcinoma patients, in line with previous reports (87). In contrast, RHOV (Ras homolog gene family member V) is a member of the Rho GTPase family that participates in regulating cytoskeletal remodeling, cell migration, and invasion (88). This study confirms that high expression of RHOV is an independent predictor of poor prognosis in lung adenocarcinoma patients, consistent with previous analyses in lung adenocarcinoma (89, 90). Moreover, MARCHF4, a membrane associated E3 ubiquitin ligase from the MARCH family, has previously been shown in prostate cancer cells to enhance survival under chemotherapeutic stress, promote an epithelial mesenchymal transition phenotype, and mitigate chemotherapy induced apoptosis (91). Notably, our study is the first to associate high MARCHF4 expression with adverse outcomes in lung adenocarcinoma.

Immune infiltration and functional analyses further demonstrated that the gene risk score based on glutathione metabolism-related genes reflects the state of the tumor immune microenvironment. Patients with high gene risk score exhibited immunosuppressive features, including diminished antigen-presenting capacity, impaired B cell and neutrophil function, and reduced tumor-infiltrating lymphocyte activity. The differential expression of immune checkpoint genes between the high- and low-risk groups underscores the link between the risk score and the tumor immune milieu. In the high gene risk score, inhibitory immune checkpoint molecules, such as LAG3, CD276, HHLA2, TNFSF9, CD70, and VTCN1, were significantly upregulated. LAG3 is an important inhibitory immune checkpoint that, by binding to MHC II molecules, suppresses T cell activation and proliferation (92). Similarly, CD276, a member of the B7 family, not only hinders the infiltration of CD8^+^ T cells into the tumor microenvironment, but also promotes T cell exhaustion by downregulating the secretion of key cytokines, such as IFN-γ (93). HHLA2, a recently identified B7 family member, hinders T cell activation, NK cell proliferation, and cytokine secretion through its interaction with the inhibitory receptor KIR3DL3 (94). The collective high expression of these inhibitory checkpoints likely establishes an immunosuppressive microenvironment that diminishes antitumor immune efficacy, leading to poorer immunotherapeutic responses and prognosis in patients with high gene risk score scores.

Using the TIDE algorithm, our study predicted immunotherapy outcomes and found that patients in the high-risk group had higher overall TIDE and immune exclusion scores, with significantly lower response and benefit rates compared to the low-risk group. These results suggest that the risk model based on glutathione metabolism-related genes not only mirrors the characteristics of the tumor immune microenvironment but is also closely linked to the efficacy of immunotherapy. Additionally, predictions via the oncoPredict algorithm revealed significant associations between the gene risk score and drug sensitivity in lung adenocarcinoma patients, providing a robust molecular basis for both immunotherapy and drug sensitivity prediction and offering new avenues for personalized treatment strategies. It is essential to acknowledge that the immunotherapy response predictions from the TIDE algorithm and the drug-sensitivity forecasts generated by the oncoPredict package in this study are exclusively outputs of in silico computational models. These results chiefly represent correlative analyses between tumor gene expression and existing datasets. Going forward, validation in real-world clinical cohorts or through *in vitro* and *in vivo* experiments will be required to establish their clinical applicability and accuracy.

Recent bioinformatics investigations have clarified the impact of glutathione metabolism on lung adenocarcinoma by constructing prognostic models based on seven genes regulating glutathione metabolism and long noncoding RNAs linked to this pathway (95, 96). By contrast, our work stratifies patients into high and low glutathione metabolic cohorts according to the metabolic heterogeneity previously observed. Building on this classification, we then identified differentially expressed genes and constructed a gene risk score. By integrating the gene risk score with tumor staging, we constructed a nomogram for prognostic prediction. This model demonstrated strong predictive performance across the TCGA training cohort and two independent GEO validation cohorts. Receiver operating characteristic curves, Kaplan–Meier survival analyses, and decision curve analysis all confirmed that the model possesses reliable predictive accuracy and clinical benefit, underscoring its potential as a valuable tool for individualized clinical decision-making in lung adenocarcinoma management.

Although this study yielded several meaningful findings, there are still some limitations. The genetic causal analysis and the construction of the prognostic model were primarily based on data from European and East Asian populations, so it remains to be determined whether the results can be generalized to other ethnic groups and broader populations. Differences in genetic backgrounds and environmental exposures may affect metabolic and immune phenotypes. Thus, future investigations should include more diverse cohorts to assess the universality of these conclusions. Moreover, our prognostic model relies solely on transcriptomic data, whereas numerous critical clinical parameters, such as pathological and imaging features, were not incorporated. Integrating additional clinical information will be essential to achieve a more comprehensive and accurate prognostic evaluation. In addition, the single-cell analysis utilized publicly available datasets. Although these datasets integrated samples from many patients, they might not fully capture the heterogeneity of the lung adenocarcinoma microenvironment. Future studies should include more patient-derived sequencing data and employ advanced techniques, such as spatial transcriptomics, to further validate and expand our understanding of the link between glutathione metabolism and immunity. Most importantly, our study is primarily based on bioinformatic analyses, and experimental validation is currently lacking. For instance, while we propose that the glutathione metabolite 5-oxoproline may promote tumorigenesis by reducing the proportion of CD28^-^CD25^++^ CD8^+^ T cells, it is necessary to confirm through animal models or *in vitro* co-culture experiments whether treatment with 5-oxoproline or modulation of glutathione metabolism can directly impair T cell function and accelerate tumor formation. Similarly, further cellular and molecular experiments are needed to validate whether key genes in the prognostic model, such as MARCHF4, can serve as potential therapeutic targets. From a translational perspective, the proposed gene risk score and prognostic model must be validated in prospective clinical trials to confirm their predictive power and clinical utility.

## 5 Conclusion

Our study reveals that elevated 5-oxoproline levels contribute to LUAD progression by impairing CD8^+^ T-cell-mediated immunity. Glutathione metabolism reprogramming is associated with immune suppression and an increased proportion of exhausted T cells in the tumor microenvironment. The identified prognostic signature provides a robust tool for predicting patient outcomes and may inform the development of targeted therapies aimed at modulating GSH metabolism and enhancing antitumor immunity in LUAD, thereby facilitating improved treatment decision-making and identification of high-risk populations requiring close follow-up.

## Data Availability

The original contributions presented in the study are included in the article/**Supplementary Material**. Further inquiries can be directed to the corresponding author.

## Supplementary material

Supplementary Figure 1 Scatter plot of the correlation between 12 core glutathione metabolism genes and the GSH score.

Supplementary Figure 2 Comparison of central carbon metabolism scores among different GSH metabolism groups. Boxplots comparing central carbon metabolism scores between different GSH metabolism groups in bulk RNA-seq for **(A)** Glycolysis/Gluconeogenesis, **(B)** Pentose Phosphate Pathway, **(C)** Citrate Cycle/TCA Cycle, and **(D)** Glycine, Serine, and Threonine Metabolism. Violin plots of metabolic scores in single-cell sequencing data for **(E)** Glycolysis/Gluconeogenesis, **(F)** Pentose Phosphate Pathway, **(G)** Citrate Cycle/TCA Cycle, and **(H)** Glycine, Serine, and Threonine Metabolism.
