# Supplementary figures and images for: Multi-omics analysis reveals glutathione metabolism-related immune suppression and constructs a prognostic model in lung adenocarcinoma
Source: Front Immunol. 2025 Jul 2;16:1608407. doi: 10.3389/fimmu.2025.1608407 (PMC12263636; doi:10.3389/fimmu.2025.1608407)

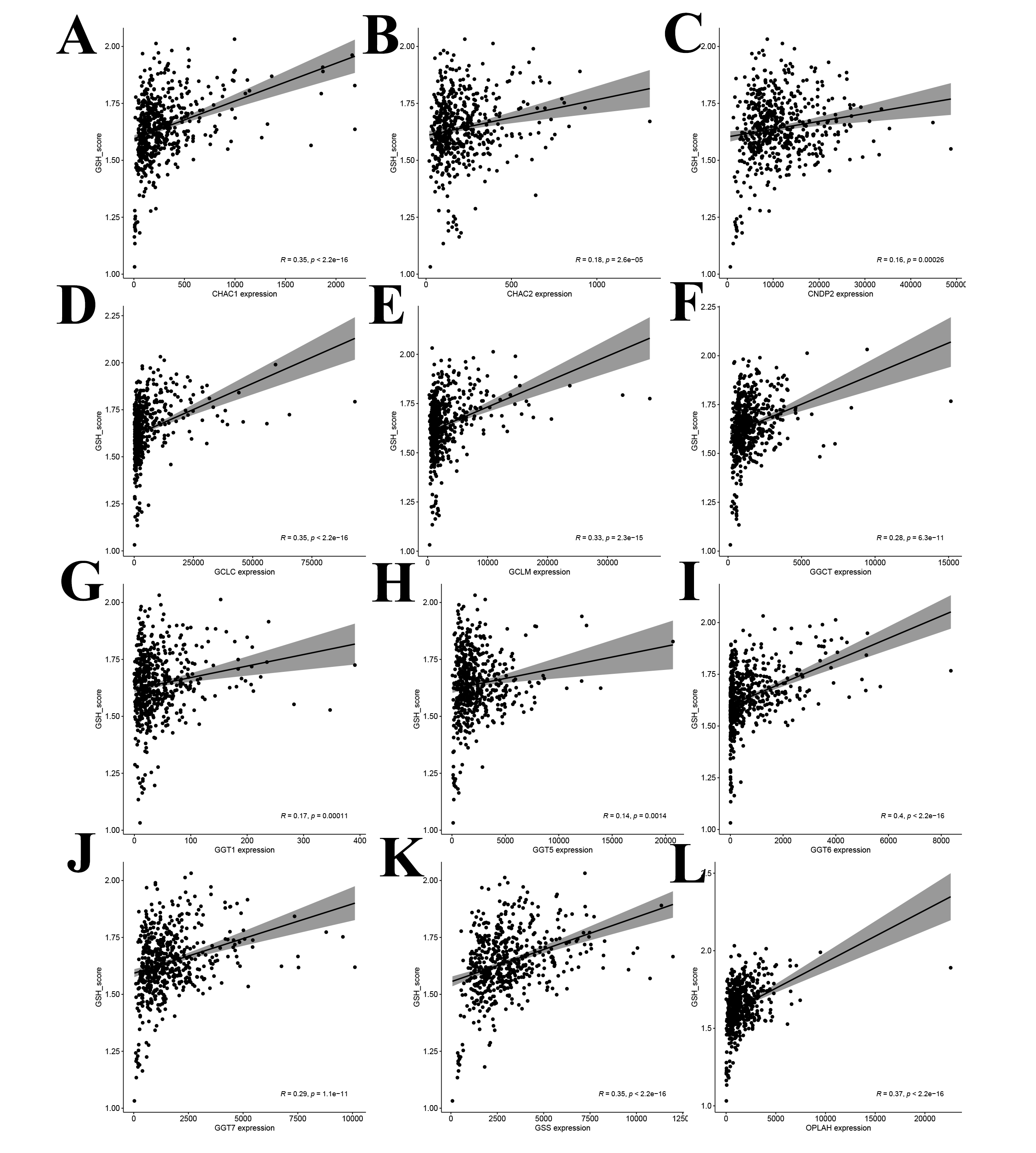

Supplement: Supplementary Figure 1 — Scatter plot of the correlation between 12 core glutathione metabolism genes and the GSH score. [file Image1.tif]

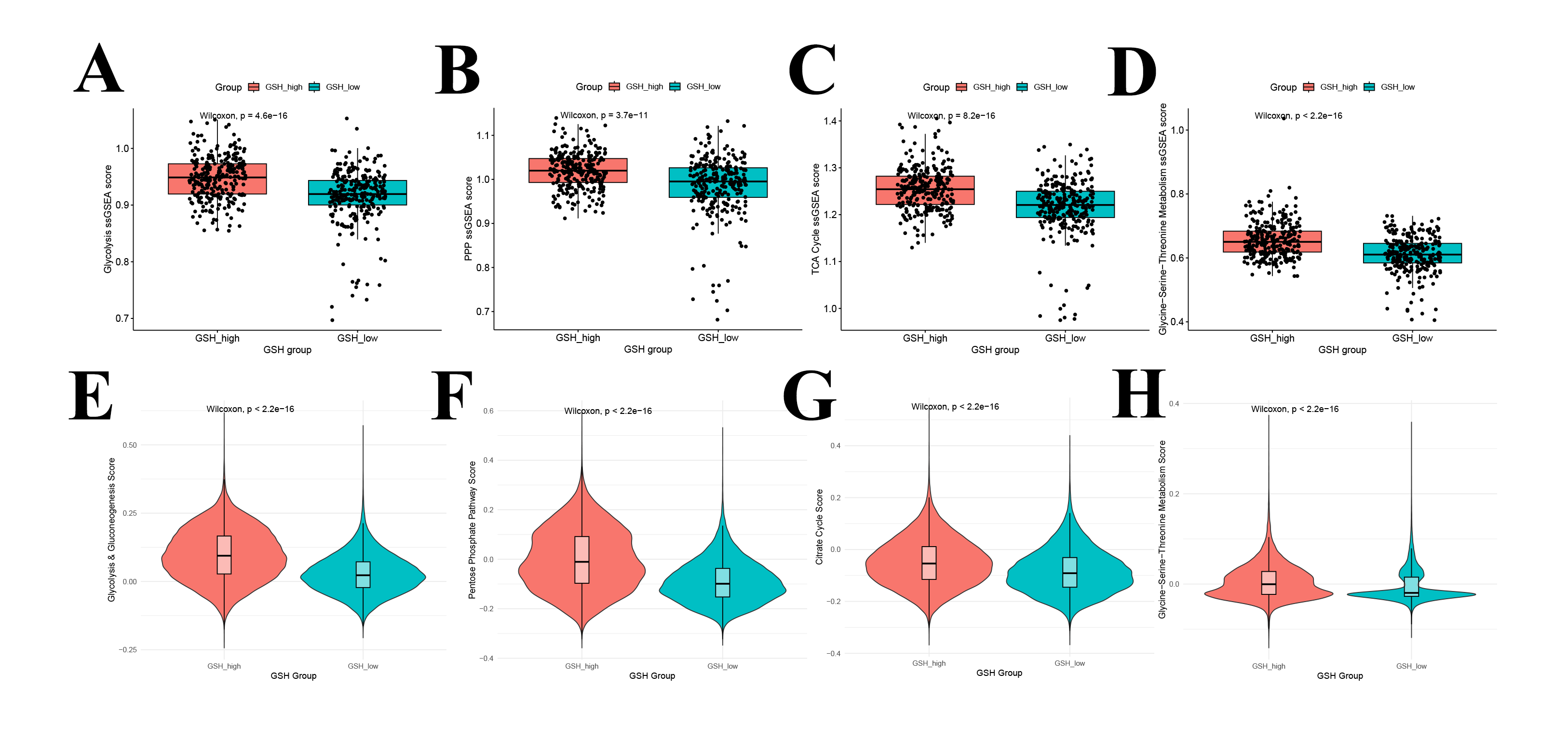

Supplement: Supplementary Figure 2 — Comparison of central carbon metabolism scores among different GSH metabolism groups. Boxplots comparing central carbon metabolism scores between different GSH metabolism groups in bulk RNA-seq for (A) Glycolysis/Gluconeogenesis, (B) Pentose Phosphate Pathway, (C) Citrate Cycle/TCA Cycle, and (D) Glycine, Serine, and Threonine Metabolism. Violin plots of metabolic scores in single-cell sequencing data for (E) Glycolysis/Gluconeogenesis, (F) Pentose Phosphate Pathway, (G) Citrate Cycle/TCA Cycle, and (H) Glycine, Serine, and Threonine Metabolism. [file Image2.tif]
