# Supplementary material for: Multi-omics analysis reveals glutathione metabolism-related immune suppression and constructs a prognostic model in lung adenocarcinoma
Source: Front Immunol. 2025 Jul 2;16:1608407. doi: 10.3389/fimmu.2025.1608407 (PMC12263636; doi:10.3389/fimmu.2025.1608407)
Supplement: Supplementary file 6 [file Table4.docx]

Table. S4 Baseline characteristics table of the study cohort

| Characteristics | TCGA | GSE31210 | GSE13213 |
| --- | --- | --- | --- |
|  | N=497 | N=226 | N=117 |
| Age |  |  |  |
| Mean | 65.4 | 59.6, | 60.7 |
| <50 | 33 (6.6%) | 23 (10.2%) | 18 (15.4%) |
| 50-60 | 90 (18.1%) | 73 (32.3%) | 25 (21.4%) |
| 60-70 | 179 (36.0%) | 123 (54.4%) | 52 (44.4%) |
| >70 | 195 (39.2%) | 7 (3.1%) | 22 (18.8%) |
| Stage |  |  |  |
| I | 277 (55.7%) | 168 (74.3%) | 79 (67.5%) |
| II | 116 (23.3%) | 58 (25.7%) | 13 (11.1%) |
| III | 79 (15.9%) |  | 25 (21.4%) |
| IV | 25 (5.0%) |  |  |
| Survival.time |  |  |  |
| Median | 664.0 | 1744.5 | 2041.0 |
| Range | 4-7248 | 221-3863 | 186-3295 |
| Survival.status |  |  |  |
| Alive | 320 (64.4%) | 191 (84.5%) | 68 (58.1%) |
| Dead | 177 (35.6%) | 35 (15.5%) | 49 (41.9%) |
